# Web-Based Isha Kriya Meditation and Breathwork in Long COVID: Mixed Methods Randomized Controlled Pilot Study

**DOI:** 10.2196/89631

**Published:** 2026-11-12

**Authors:** Akila Rayapuraju, Enas Mohamed, Preeti Upadhyay Reed, Hibiki Orui, Morgan Pratt, Nashaw Jafari, Tulsi Chase, Amberly Ticotsky, Jason Maley, Balachundhar Subramaniam

**Affiliations:** 1 Sadhguru Center for a Conscious Planet, Department of Anesthesia, Critical Care and Pain Medicine,, Beth Israel Deaconess Medical Center, 1 Brookline Place, Unit 621, Brookline, MA, 02445, United States, 1 617-667-3112; 2 Harvard Medical School, Boston, MA, United States; 3 Critical Illness and COVID-19 Survivorship Program, Department of Pulmonary, Critical Care and Sleep, Beth Israel Deaconess Medical Center, Boston, MA, United States; 4 Sionna Therapeutics, Waltham, MA, United States

**Keywords:** long COVID, meditation, mental health, telemedicine, dyspnea

## Abstract

**Background:**

Long COVID affects millions of adults, causing fatigue, dyspnea, cognitive symptoms, and mood disturbances. Effective treatments are limited, and many patients cannot access multidisciplinary rehabilitation because of symptom fluctuation, distance, or specialist shortages. Web-based mind-body interventions may offer accessible, scalable support alongside existing care.

**Objective:**

We assessed the feasibility and efficacy of a 3-week web-based yogic breathing and meditation program for long COVID symptoms.

**Methods:**

In this completed, parallel-group, 6-week waitlist-controlled, mixed methods randomized trial, adults aged 18‐70 years with physician-confirmed long COVID were recruited from US clinics and self-referral and randomized 1:1 to immediate intervention or waitlist control, crossing over at week 3. The program included live webinars and video guides for daily self-practice. The primary outcome was adherence, defined as practicing at least 1 of 3 techniques on ≥4 days/week across all 3 active-intervention weeks. Secondary outcomes included perceived stressmood, quality of life, dyspnea, and somatic symptom burden, assessed at baseline, 3 weeks, and 6 weeks. Quantitative outcomes were analyzed using linear mixed-effects models. Semistructured interviews and focus groups underwent conventional content analysis and were integrated with quantitative findings via joint display.

**Results:**

Of 189 randomized participants, 1 was excluded post-randomization for a protocol deviation; 188 (96 intervention, 92 waitlist) began the study. Of these, 124 reached the active intervention period. Among 58 with complete adherence data, 55% met the adherence threshold (60% intervention, 50% waitlist). Secondary analyses included 185 participants with ≥1 assessment. No adverse events were reported. Compared with waitlist control, the intervention group showed greater reductions in total mood disturbance (MD=−10; 95% CI −19 to −1.6; P=.02), negative mood (MD=−7.5; 95% CI −15 to −0.44; P=.04), fatigue (MD=−2.9; 95% CI −4.6 to −1.2; P=.001), MDP immediate perception (MD=−5.5; 95% CI −9.4 to −1.6; P=.007), MDP sensory dimension (MD=−5.1; 95% CI −8.5 to −1.7; P=.003), and somatic symptom burden (MD=−2.0; 95% CI −3.6 to −0.38; P=.02), and greater increases in positive mood (MD=2.9; 95% CI 0.66 to 5.1; P=.01) and vigor (MD=1.7; 95% CI 0.18 to 3.2; P=.03). Stress and quality of life did not differ significantly. Qualitative findings identified medical-system struggles, digital delivery, online support, and practices’ effects on resilience and identity. Integration showed convergence for dyspnea and mood, divergence for stress and quality of life, and expansion through themes of medical-system experience, identity, and social connection.

**Conclusions:**

This web-based yogic breathing and meditation program appears feasible and accessible for people with long COVID. By combining 3 practices in a brief protocol and integrating quantitative and qualitative evidence, this study provides novel insight into both symptom outcomes and dimensions of recovery not captured by standardized measures. Further evaluation should assess longer-term effects and integration into scalable telehealth and virtual rehabilitation pathways.

## Introduction

### Background and Rationale

Since the COVID-19 pandemic began in 2020, long COVID has affected 16% or an estimated 18 million US adults and remains a substantial global burden [1-5]. For several years, the condition lacked a clear definition due to its multifaceted nature and more than 200 reported symptoms, contributing to misdiagnosis and limited treatment options [2]. In 2024, the National Academies of Sciences, Engineering, and Medicine (NASEM) defined long COVID as an “infection-associated chronic condition” that persists for at least 3 months and affects one or more organ systems [6]. More recently, a 2026 meta-analysis found long COVID symptoms persist for 6 months or longer and fluctuate in frequency and intensity over time, characterizing long COVID not as a fixed syndrome but a dynamic condition. The most prevalent symptoms include fatigue, brain fog, dyspnea, and sleep impairment [7]. In addition, individuals with long COVID report worse mental health outcomes and greater loneliness compared to the general population, underscoring a critical need for greater mental health and social support structures [8].

Despite its emergence as a chronic condition, its long-term physical and neuropsychiatric consequences, and its continued global prevalence, accessible treatments for long COVID remain limited due to a lack of robust evidence evaluating both pharmacological and nonpharmacological treatments [3,9,10]. Rehabilitation programs can be helpful but require careful monitoring, while pharmacological therapies typically target only specific symptoms [10]. Multidisciplinary, tailored approaches are therefore needed to address the interacting biological and psychosocial processes underlying long COVID [7,10,11]. In addition, a 2025 meeting report released by the US National Institute of Allergy and Infectious Diseases emphasized the importance of minimizing patient burden and expanding at-home, digitally delivered treatment options to accommodate fatigue, cognitive, and mobility-based limitations [2]. These priorities highlight the need for digital and scalable low-burden interventions.

Mind-body interventions (MBIs), such as yoga and meditation practices, integrate mind, body, brain, and behavior and offer a promising nonpharmacological approach. MBIs have generally been associated with improved well-being and reduced anxiety and depression across chronic conditions, while breathing and meditation practices have shown benefits for pulmonary function and emotional regulation, including in the context of COVID-19–related stress [12-16]. Specifically, yogic breathing practices have been shown to regulate autonomic function, enhance vagal tone, and strengthen interoceptive awareness [17,18]. Brief, guided practices such as Isha Kriya, Nadi Shuddhi, and Simha Kriya have been associated with reductions in acute stress and mood disturbance, with regular practice of Isha Kriya showing potential benefits for anxiety and depression [19-23]. In addition, the rapid expansion of mindfulness applications and online courses has made digital MBIs widely accessible, positioning them as practical options for individuals whose symptoms make in-person care difficult [24].

It is also important to emphasize that long COVID is a physiological condition. When it is instead treated as primarily psychological, this misattribution can compound the stigma, dismissal, and gaslighting many patients already face, on top of significant impacts on family roles, employment, and quality of life [25]. These combined physical and psychosocial stressors may also contribute to the anxiety, mood disturbance, and stress reported in this population. Web-based MBIs are therefore proposed not as a stand-alone treatment, but as a complementary component of larger tailored approaches, potentially supporting both physiological symptoms, such as breathing difficulty, and the mental health burden associated with living with long COVID.

### Objectives

This randomized controlled trial evaluated the feasibility and effectiveness of the Breathing and Wellness program, which includes web-based guided meditation and breathing practices, over 3 weeks. We hypothesized that the Breathing and Wellness program would be feasible and would lead to improvements in stress, mood, dyspnea, quality of life, and somatic symptom burden.

## Methods

### Trial Design

This study was a waitlisted, parallel-group, mixed methods randomized controlled trial (RCT) among individuals with long COVID conducted at Beth Israel Deaconess Medical Center (BIDMC) with an intervention and control arm. The qualitative component was added after trial commencement to complement quantitative data by exploring participants’ lived experiences and providing contextual understanding of the intervention’s delivery and effects. Integration occurred during the interpretation phase through a joint display approach, which facilitated the identification of convergence, divergence, and expansion across outcomes. Results are reported in accordance with the CONSORT (Consolidated Standards of Reporting Trials) 2025, CONSORT E-HEALTH (Consolidated Standards of Reporting Trials of Electronic and Mobile Health Applications and Online Telehealth; version 1.6.1), GRAMMS (Good Reporting of a Mixed Methods Study), and COREQ (Consolidated Criteria for Reporting Qualitative Research) guidelines [26-29]. Patients and members of the public were not involved in the design, conduct, reporting, or dissemination plans of this study. The trial protocol can be accessed in the Multimedia Appendix 1.

After enrollment, intervention group participants received a link to sign up for the webinars on Acuity, a Health Insurance Portability and Accountability Act (HIPAA)–compliant online scheduling platform. Detailed instructions about how to prepare for the class and the webinar Zoom (Zoom Communications, Inc) link were emailed to participants immediately after signing up. Reminder emails to attend the class were automatically sent through Acuity at 48 hours, 24 hours, and 2 hours before the session. No reminders explicitly prompting daily practice were sent. Class attendance was tracked by instructors in a spreadsheet accessible to a research staff member for cross-verifying self-reported attendance.

Intervention group participants completed weekly surveys from baseline through 3 weeks and once again at 6 weeks. The waitlisted control group was instructed to continue their daily routine until 3 weeks after baseline, when they received an email with an Acuity link to sign up for their first webinar. Waitlisted participants completed weekly surveys for all 6 weeks. Both groups received adherence surveys weekly during the active intervention period. Randomization, surveys, and automated survey reminders were implemented via REDCap (Vanderbilt University), an electronic data collection platform. Participants were followed up via email if they missed completing any questionnaire.

In addition, consenting participants from both groups took part in semistructured interviews and focus groups conducted via Microsoft Teams. Intervention group interviews were held 3 weeks after completion of the Breathing and Wellness program, while waitlist group interviews occurred prior to starting the intervention. Individual interviews and focus group discussions (FGDs) lasted 60 to 90 minutes, during which interviewers checked in regularly to ensure participants’ comfort and accommodate fatigue.

### Eligibility Criteria

Participants were recruited on a rolling basis through referrals by long COVID clinics (including the BIDMC Critical Illness and COVID-19 Survivorship Program and the Penn Medicine Post-COVID Assessment and Recovery Clinic) and online self-referrals (eg, via flyers shared in long COVID support groups) until the minimum sample size was reached. US residents proficient in English between 18‐70 years of age with polymerase chain reaction (PCR)-confirmed COVID-19 history and a physician-reviewed long COVID diagnosis were eligible for the study. Although computer and internet literacy were not explicit eligibility criteria, both were required to engage with the intervention.

Individuals with long COVID were defined as those with a history of COVID-19 infection, occurrence of illness around 3 months after COVID-19 onset, and persistent symptoms for at least 2 months not explained by an alternative diagnosis as per the 2021 World Health Organization (WHO) consensus definition [30]. This definition was selected because the study was designed and initiated in 2021, at which time the WHO definition was among the most widely accepted. No minimum symptom-severity threshold was imposed for study enrollment, so baseline symptom severity varied across participants. Participants with severe psychiatric conditions (major depression, bipolar disorder, and schizophrenia) were excluded as active symptoms could confound self-reported mood and stress outcomes and affect capacity for daily self-practice. Ineligible participants could still sign up for the Breathing and Wellness program to learn the practices.

A nested sampling approach was used for qualitative study procedures. The qualitative sample was drawn from participants enrolled in the main trial and therefore followed the same eligibility criteria. Participants were recruited on a rolling basis from both the intervention and waitlist control groups. Eligible participants were contacted by telephone and invited to take part in interviews conducted via Microsoft Teams, exploring their experiences living with Long COVID and interactions with physicians and family members. No participants refused to participate or dropped out of the qualitative component. Recruitment continued until thematic saturation was achieved. Participant identity was verified using a full name and confirmed email address to prevent duplicate enrollment. No individuals other than the participants and researchers were present during the interviews or FDGs.

### Intervention

Three practices were delivered synchronously on Zoom through 60-minute breathing and wellness webinars led by a trained instructor. Simha Kriya is a 3-minute restorative breathing practice with rapid, deep breathing and breath retention designed to improve pulmonary function. Nadi Shuddhi is a 4-minute alternate-nostril breathing technique designed to stabilize and balance the body and mind. Isha Kriya is a 15-minute guided meditation practice incorporating focus, sound, and breathing synchronized with one thought. As each practice individually has been shown to enhance overall mental health and regulate mental and physical stress, we hypothesized that the effects could compound when combined [15,19,22,23]. When completed together, practice time was approximately 30 minutes.

Participants were required to attend at least one webinar to learn the practices, followed by weekly optional but highly encouraged review sessions for a total of 3 sessions offered over the 3-week active intervention period. The webinars followed a hybrid model, combining live instruction with prerecorded demonstration videos. Webinar participation required a device with internet access, a camera, and a microphone to join Zoom sessions. Demonstration videos were provided by email postwebinar for daily self-practice and are available on YouTube. Links have been provided in Multimedia Appendix 2.

Instructors observed participants during sessions and offered individualized corrections for participants experiencing persistent difficulty. All webinars followed an identical, standardized script regardless of instructor or session date, and no modifications were made to the intervention protocol during the study. There were no cointerventions provided in addition to the breathing and meditation practices.

Instructors had 1750 hours of training certified by Isha Hatha Yoga School and completed an additional structured ~7‐8-week training program, incorporating supervised practice teaching and a standardized competency evaluation that they were required to pass before teaching independently. Training included a long COVID–specific curriculum addressing symptom variability, individualized accommodation, and a structured pathway for escalating participant symptoms or questions to the study’s medical co-investigators. Full details of the training program structure and curriculum content are provided in Multimedia Appendix 3.

### Randomization and Blinding

Participants were randomized by a study team member into either the intervention or waitlist group using the 1:1 block randomization technique on REDCap. Only the statistician had access to the random allocation sequence as they prepared it. Only webinar instructors were masked to study group allocation.

### Outcomes

#### Feasibility

Feasibility was assessed across four domains, each evaluated using an analytic population matched to the question being asked: (1) recruitment (referral and enrollment yield, assessed among all referred and screened participants), (2) retention (proportion of randomized participants remaining enrolled at each study timepoint, assessed among all 188 randomized participants), (3) adherence (proportion of participants meeting the prespecified practice threshold, assessed among the 124 randomized participants who reached the active intervention period and received at least one adherence survey—see questions in Multimedia Appendix 4), and (4) acceptability (participants’ qualitative appraisals of the program). Adherence served as the primary outcome.

The practice threshold was defined as completing at least 1 of the 3 intervention practices (Isha Kriya, Nadi Shuddhi, and Simha Kriya) on at least 4 days per week, in all 3 of a participant’s active-intervention weeks. This frequency-based threshold was adopted from prior trials of the same or related Isha practices in healthy populations [22,31,32].

#### Perceived Stress Scale

Perceived stress was defined as the key secondary outcome and assessed at baseline, week 3, and week 6. This widely used and validated 10-item scale was designed to quantify how often individuals experienced life events as stressful or overwhelming over the past month on a scale from 0 (Never) to 4 (Very Often). Higher Perceived Stress Scale (PSS) scores are associated with elevated perceptions of stress [33]. PSS has been used previously to assess perceived stress in populations with long COVID, where those with long COVID had higher odds of moderate to high stress (≥14) [8].

#### Profile of Mood States

Mood states were assessed using the validated 40-item (abbreviated version) Profile of Mood States (POMS) scale, which measures mood across seven dimensions: tension, anger, fatigue, depression, vigor, confusion, and esteem. A range of adjectives describing emotional experiences are rated on a 5-point scale from “Not At All” to “Extremely.” Results can be categorized into total mood states, positive moods, and negative moods. A higher total mood disturbance (TMD) score indicates greater mood disturbance [34]. POMS has not previously been used to assess mood states in populations with long COVID.

#### Quality of Life (12-Item Short Form Survey)

Quality of Life was assessed using a validated 12-item scale spanning 8 domains: physical functioning, role physical, bodily pain, general health, vitality, social functioning, role emotional, and mental health. Results are categorized into the physical component summary (PCS) and mental component summary (MCS). Scores above 50 indicate better-than-average health and below 50 indicate below-average health [35]. The 12-Item Short Form Survey (SF-12) has been previously used to assess quality of life in populations with long COVID [36].

#### Multidimensional Dyspnea Profile

The sensory and emotional components of breathlessness were assessed using the validated scale, Multidimensional Dyspnea Profile (MDP). Only the “immediate perception domain” was assessed in this study, consisting of the 5 sensory qualities (physical breathing effort, air hunger, tightness, mental breathing effort, and hyperpnea) and overall breathing discomfort. Overall breathing discomfort was rated on a scale of “neutral” to “unbearable” when the individual last climbed a flight of stairs or went for a short walk. Intensity of the 5 sensory qualities was assessed on a scale from “0” (“none”) to “10” (“as intense as I can imagine”) [37]. The MDP has been used previously to assess dyspnea in populations with long COVID [36].

#### Somatic Symptoms Scale

Burden of common physical symptoms was assessed using the validated 8-item Somatic Symptoms Scale (SSS-8), previously used in populations with long COVID [38] (Multimedia Appendix 5 for survey items). Symptoms are rated on a scale from “0” (“not at all”) to “4” (“All the time”). Scores range from 0 to 32, with higher scores indicating a greater somatic symptom burden [39]. SSS-8 has been used previously to assess somatic symptom burden in populations with long COVID [38].

#### Qualitative Interviews

Semistructured individual interviews explored participants’ prognosis, symptom severity, available resources, daily impacts, coping strategies, and their interactions with physicians and family members. Additionally, those in the intervention group reflected on their experiences with the breathing and meditation practices, and the accessibility and usability of the program as a digital health tool. FGDs used a separate interview guide designed to explore collective reflections, shared experiences, and group dynamics, complementing individual interviews (Multimedia Appendix 6).

Interviews were conducted by a postdoctoral research fellow with a master’s degree in social sciences and global health delivery and prior training in qualitative research, including conducting interviews and data analysis. The interviewer’s interest in the study was to understand participants’ lived experiences with long COVID and had not personally practiced the intervention techniques. The interviewers had no prior relationship with participants beyond their role in the study. Participants were aware that the interviewer was a member of the research team and were informed of the purpose of the study and interview topics. The interview guides were not formally pilot tested. All interviews were conducted in English and were video and audio-recorded with consent. No repeat interviews were conducted. Field notes were taken during both the individual interviews and FGDs.

### Sample Size

Because feasibility trials are not typically powered for clinical outcomes, and adherence was the primary endpoint, the sample size was determined based on our key secondary outcome, the PSS. Power calculations indicated that at least 68 participants (34 per group) were required. An interim analysis of the primary endpoint was planned when 50% (34/68) of participants were randomized and completed the 3-week follow-up, to be conducted by an independent statistician blinded to treatment allocation. The trial was not designed to stop for futility, except at the discretion of the institutional review board (IRB) during safety monitoring.

### Statistical Methods

#### Missing Data

Given substantial attrition relative to sample size, multiple imputation was judged likely to introduce instability and false precision. Little missing completely at random (MCAR) test assessed whether missingness in weekly adherence data and outcome measures was related to observed values, with randomization arm included as an auxiliary variable (Results). Outcome analyses used linear mixed-effects models (LMMs) with maximum likelihood estimation, which provides valid inference under the missing-at-random assumption. To bound the impact of missing adherence data on the adherence rate and its association with outcome change, both were computed under a worst-case (all missing weeks assumed nonadherent) and best-case (all missing weeks assumed adherent) scenario.

#### Feasibility

All feasibility indicators were reported as frequency and percentage of participants. Recruitment and retention numbers were based on all 188 randomized participants. Adherence assessments were based on participants who reached the active intervention period and received at least one adherence survey (n=124).

#### Efficacy

Repeated-measures analysis of secondary outcomes used an LMM with random intercepts for individuals, maximum likelihood estimation, and fixed effects for group, time, and a group×time interaction, comparing intervention to waitlist control from baseline to Week 3. Participants with only baseline data contributed to intercept but not slope estimation. All randomized participants with ≥1 outcome assessment (n=185). Three were excluded as they had no outcome data at any time point.

#### Post Hoc Sensitivity Analyses

Alternative thresholds (≥3 and ≥5 days/week, same all-3-weeks requirement) were examined as sensitivity analyses on the adherence definition. Baseline demographic and clinical characteristics were compared descriptively between retained and nonretained participants, and between adherent and nonadherent participants, using the Fisher exact test (categorical variables) and 2-sided Welch *t* tests (continuous variables), to assess whether retention was associated with baseline severity. An LMM evaluated the overall intervention effect among all participants (intervention and waitlist control groups) who completed the active intervention period (n=155). A post hoc per-protocol analysis was also conducted, restricting the primary model to participants completing the study through Week 6. Finally, to further examine the relationship between adherence and secondary outcomes, we compared secondary outcomes for adherent and nonadherent participants under complete-case, worst-case, and best-case assumptions using the same mixed-effects specification as the main LMM. We also modeled the adherence-outcome association as a continuous gradient of practice frequency to assess dose-response. These analyses are reported as hypothesis-generating, since adherence status is determined postrandomization and associations should not be interpreted causally. Results can be found in Multimedia Appendices 3 and 7.

All models were adjusted for age, gender, race, and ethnicity, reported as adjusted mean differences with 95% CIs, *P* values, and partial eta-squared (small ≥.01, medium ≥.06, and large ≥.14). All analyses were performed in R (version 4.4.1; R Foundation for Statistical Computing).

### Qualitative Analysis

Conventional content analysis was conducted on transcriptions of thirty participants who completed individual interviews (15 intervention and 15 waitlist participants). In addition, 2 FGDs were conducted. The FGD followed a separate semistructured discussion guide designed for a group setting. Although the guide differed from those used in the individual interviews, both addressed the same overarching research objectives and yielded conceptually overlapping data. Consequently, data from both sources were analyzed together using a unified inductively developed codebook, enabling consistent coding and comparison across individual interview and FGD data [40].

Variability in interview length reflected the level of detail participants chose to share, but both shorter and longer interviews yielded rich data. Transcripts were reviewed against the audio recordings for accuracy. Transcripts were not returned to participants for comment or correction. An initial codebook was generated inductively from both the individual interview and FGD transcripts, refined through iterative team discussions, and then applied to all transcripts in Dedoose (v9.0.107) by 3 researchers (EM, NJ, and AR). Using a unified codebook ensured analytic consistency across individual and group data while enabling meaningful comparison and integration of findings across both data sources. The researcher had prior training and experience in qualitative research, including developing semistructured interview guides, conducting qualitative interviews, coding qualitative data, and performing qualitative data analysis.

We monitored thematic saturation throughout coding. No new codes emerged by approximately the 12th interview in each group. Final interviews and FGD confirmed that saturation had been reached. Three analysts (EM, NJ, and AR) collaboratively examined the coded data to derive themes representing participants’ experiences, and all co-authors reviewed and validated the results to ensure accuracy and consistency. Participants were not asked to provide feedback on the qualitative findings. The team also practiced reflexivity by discussing how their perspectives might influence coding and interpretation, ensuring that themes remained grounded in participants’ accounts.

### Mixed Methods Integration

Following independent analyses of the quantitative and qualitative datasets, findings were integrated using a convergence coding matrix and joint displays. Quantitative outcomes and qualitative themes addressing similar constructs were compared across key domains, including respiratory symptoms and psychological well-being, functional recovery, quality of life, and experiences of the digital rehabilitation program. Findings were classified as “convergent” when both datasets supported similar conclusions for the same construct, “divergent” when they differed in direction or emphasis, and “expansive” when qualitative findings provided insights beyond those captured by the quantitative measures. Three analysts (EM, NJ, and AR) independently reviewed and classified the integrated findings, with any differences in interpretation discussed until consensus was reached before finalizing the joint displays and meta-inferences. The joint displays facilitated systematic comparison of the datasets and informed the development of integrated meta-inferences, allowing the qualitative findings to explain, contextualize, and extend the quantitative results while preserving the integrity of each dataset. No qualitative themes directly contradicted the quantitative findings.

### Ethical Considerations

Ethical approval was given by the Institutional Review Board at BIDMC in Boston, Massachusetts (2021P000552). All participants provided electronic informed consent via REDCap. Participants received a US $25 gift card upon completion of the study. Study data have been deidentified. No images of individual participants were included in the manuscript or supplementary material, and therefore identification of participants is not possible.

### Harms

Given the psychological nature of the assessments and interviews, a safety monitoring plan was in place: a licensed clinician was available to respond immediately to any participant expressing suicidal ideation or thoughts of self-harm, with referral to emergency care as needed.

### Trial Registration

The trial was retrospectively registered at ClinicalTrials.gov (NCT05139979) on September 23, 2021, 6 days after enrollment of the first participant. The delay reflects administrative processing time rather than delayed intent to register, and the trial’s registered outcome measures are unchanged from registration to this manuscript.

### Deviations From Protocol

One participant randomized to the waitlist control group was excluded after randomization but prior to the initiation of any study procedures beyond the baseline survey. Study staff discovered that this participant had previously attended intervention webinars prior to study enrollment, a violation of eligibility. This participant is included in the total randomized count (189) but excluded from all subsequent analyses.

## Results

### Participant Flow

Between September 15, 2021, and February 7, 2024, 758 participants were referred to the study, and 557 were assessed for eligibility. About 369 ineligible participants were excluded from the study, leaving 189 randomized and 188 allocated to either the intervention (n=96) or waitlist group (n=92). Participant flow, including reasons and timing of attrition and interview flow, is presented in Figures 1 and 2.

**Figure 1.**
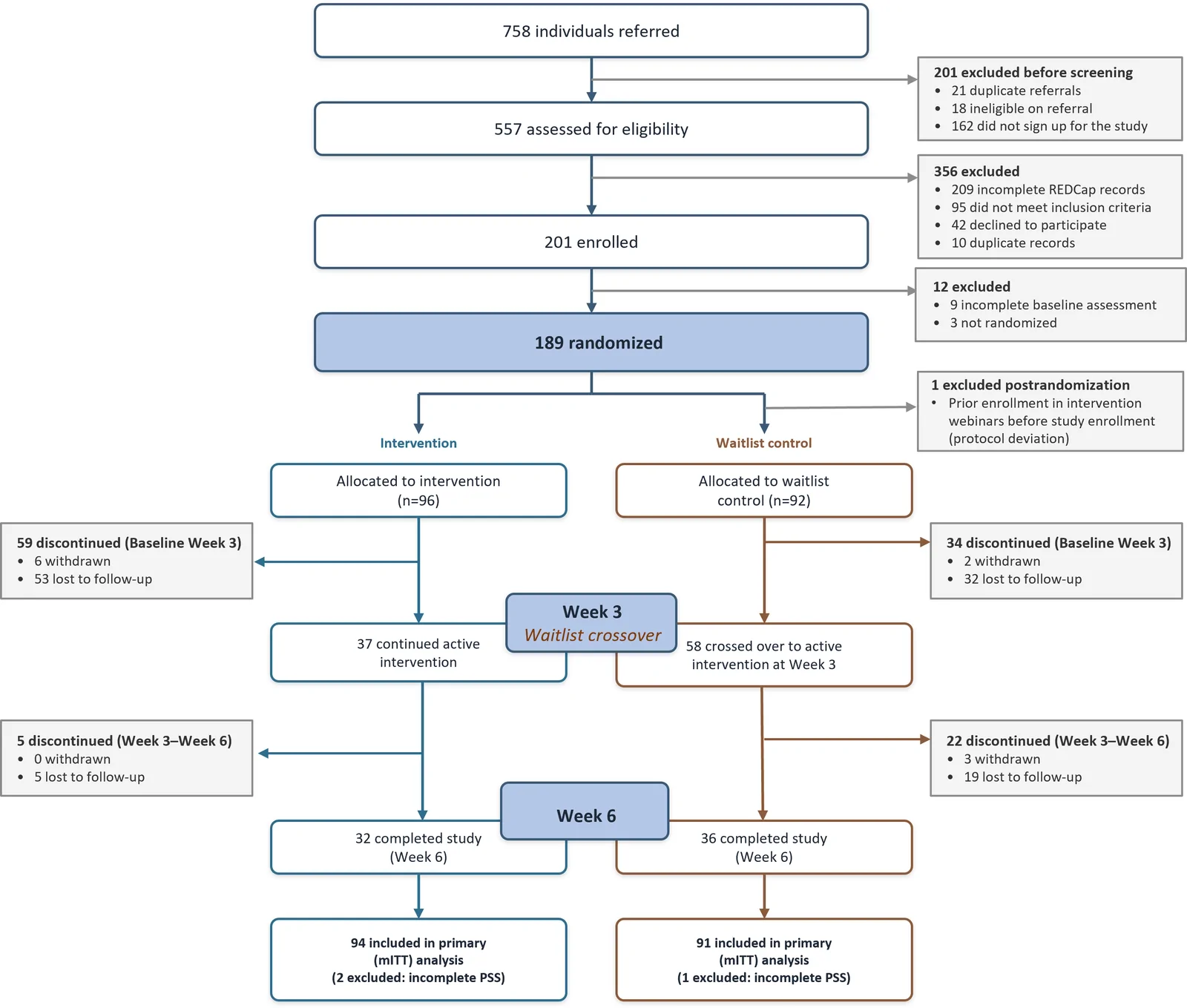
CONSORT (Consolidated Standards of Reporting Trials) participant flow diagram. mITT: modified intention-to-treat; PSS: Perceived Stress Scale.

**Figure 2.**
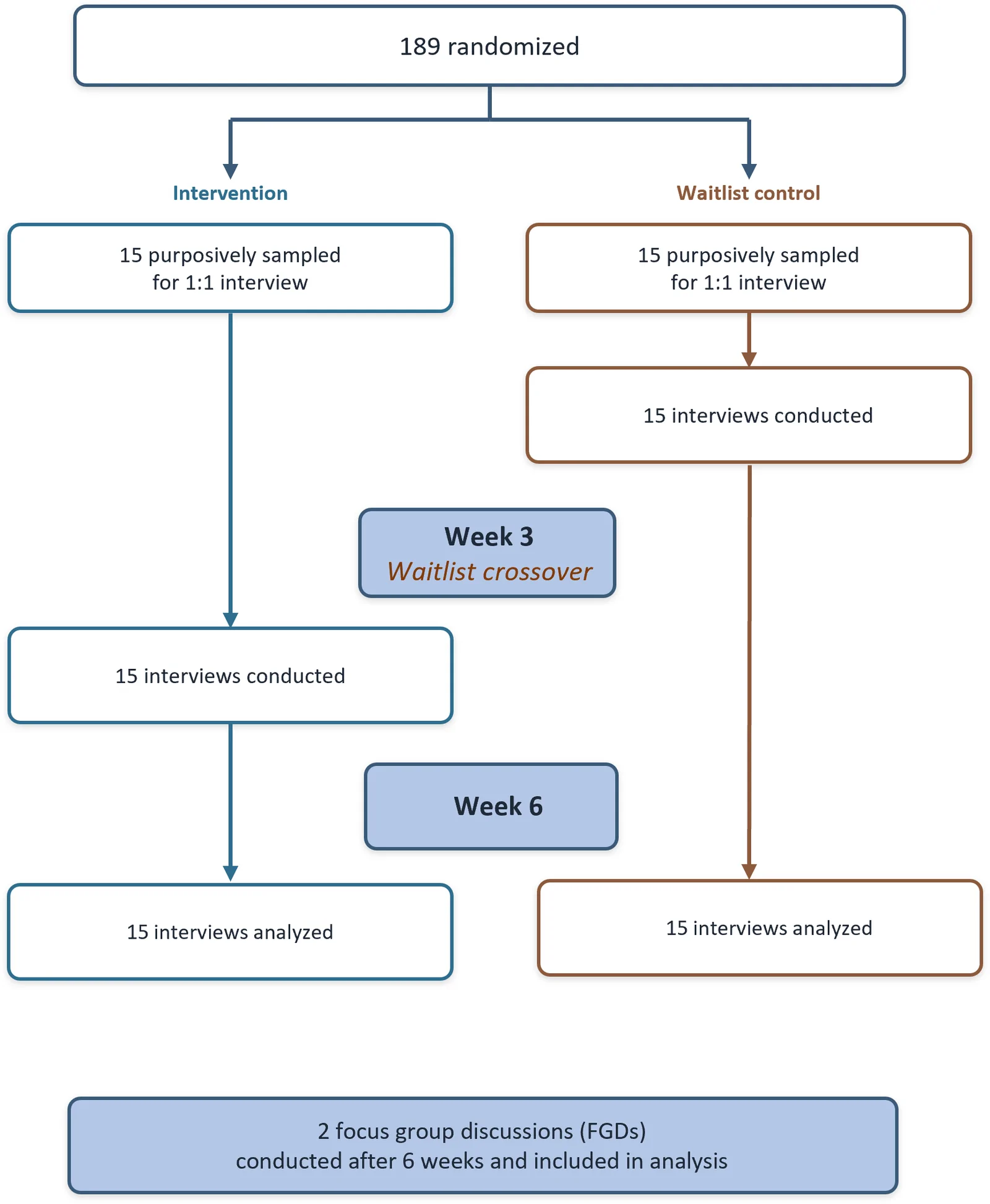
Qualitative interview participant flow. FGD: focus discussions group.

The study groups were well-matched on demographic measures: 56 (30%) participants were aged 40‐49 years, 146 (78%) were female, and 160 (85%) were non-Hispanic and White (Table 1). Education, socioeconomic status, and digital or computer literacy were not formally assessed. The intervention and waitlist groups had comparable baseline scores across all secondary outcomes. Both groups showed poor quality of life (PCS12 and MCS12 <50) and moderate stress at baseline, with little change by week 3 (Table S1 in Multimedia Appendix 3).

**Table 1.** Baseline characteristics of participants with long COVID (September 2021-February 2024).

Characteristics	Participants, n (%)		
	Overall (n=188)	Intervention (n=96)	Waitlist (n=92)
Age (years)			
18‐29	18 (10)	11 (11)	7 (7.6)
30‐39	44 (23)	24 (25)	20 (22)
40‐49	56 (30)	29 (30)	27 (29)
50‐59	41 (22)	20 (21)	21 (23)
60‐69	27 (14)	12 (13)	15 (16)
70+	2 (1)	0 (0)	2 (2)
Sex			
Female	146 (78)	72 (75)	74 (81)
Male	38 (20)	23 (24)	15 (16)
Other	3 (2)	1 (1)	2 (2)
Missing	1 (1)	0 (0)	1 (1)
Race and ethnicity			
non-Hispanic White	155 (82.4)	79 (82.3)	76 (82.6)
Hispanic White	3 (1.6)	3 (3.2)	0 (0)
Hispanic, other race	4 (2.2)	2 (2.1)	2 (2.2)
White, ethnicity not reported	3 (1.6)	2 (2.1)	1 (1.1)
Black or African American	7 (3.8)	3 (3.2)	4 (4.4)
Asian	9 (4.9)	4 (4.3)	5 (5.5)
Multiracial	1 (0.5)	0 (0)	1 (1.1)
Other	2 (1.1)	0 (0)	2 (2.2)
Missing	4 (2.2)	3 (3.2)	1 (1.1)

### Quantitative Results

#### Feasibility

##### Recruitment

Around 758 participants were referred to the study, and 557 were assessed for eligibility; 369 were excluded as ineligible, leaving 189 enrolled and 188 randomized (Figure 1), indicating strong recruitment demand for the program.

##### Retention

Of 188 participants randomly allocated to a study group, 68 (36%) had outcome data by week 6: 32 in the intervention arm and 36 in the waitlist arm.

##### Adherence

Adherence was defined as completing at least one intervention practice (Isha Kriya, Nadi Shuddhi, or Simha Kriya) on ≥4 days/week in all 3 of a participant’s active-intervention weeks. Participants missing data in any of the 3 weeks could not be classified against this threshold and were treated as missing rather than defaulted to nonadherent. This was assessed among the 124 participants who reached the active intervention period and received at least one adherence survey. Of these, 9 (7%) withdrew from the study before completing their required surveys, and 57 (46%) remained enrolled but had incomplete or entirely missing adherence data. Missing adherence data among enrolled participants was concentrated disproportionately in the waitlist arm relative to the intervention arm (Table 2, Panel A). Among the 58 participants with complete 3-week adherence data, 55% (32/58) met the adherence threshold: 60% (18/30) in the intervention arm and 50% (14/28) in the waitlist arm (Table 2, Panel B).

**Table 2.** Adherence data completeness and adherence outcome. Adherence was defined as completing at least 1 of 3 practices on ≥4 days/week in all 3 active-intervention weeks. The active intervention period was baseline – week 3 for the intervention group and week 3-week 6 for the waitlist control group.

Categories	Intervention, n/N (%)	Waitlist, n/N (%)	Total, n/N (%)
Panel A: data completeness among participants who reached the active intervention period and received at least one adherence survey			
Complete 3-week data	30/47 (63.8)	28/77 (36.4)	58/124 (46.8)
Incomplete data, retained^a^	12/47 (25.5)	6/77 (7.8)	18/124 (14.5)
No response, retained^b^	0/47 (0)	39/77 (50.6)	39/124 (31.5)
Withdrawn^c^	5/47 (10.6)	4/77 (5.2)	9/124 (7.3)
Panel B: adherence outcome, among participants with complete adherence data in all three active-intervention weeks			
Nonadherent	12/30 (40.0)	14/28 (50.0)	26/58 (44.8)
Adherent	18/30 (60.0)	14/28 (50.0)	32/58 (55.2)

a Retained and continued to receive adherence surveys; responded to only 1-2 of 3 required weeks.

b Retained and continued to receive adherence surveys; did not respond to any of the 3 required weeks.

c Discontinued the study; did not receive adherence surveys for the remainder of the active intervention period.

Little MCAR test was nonsignificant for the weekly adherence data overall (*χ*²_9_=8.51; *P*=.48) and by arm but significantly related to arm when arm was included as a variable (*χ*²_16_=54.2; *P<*.001), consistent with a missing-at-random rather than MCAR mechanism. To account for missingness, we bounded the true adherence rate under best-case and worst-case missing-data assumptions. Adherence ranged from 38.3% (18/47) to 55.3% (26/47) in the intervention arm and 18.2% (14/77) to 75.3% (58/77) in the waitlist arm (Figure 3), reflecting the higher proportion of missing adherence data in the waitlist arm. Individual and mean adherence trajectories across the 3 active-intervention weeks are shown in Figure 4.

**Figure 3.**
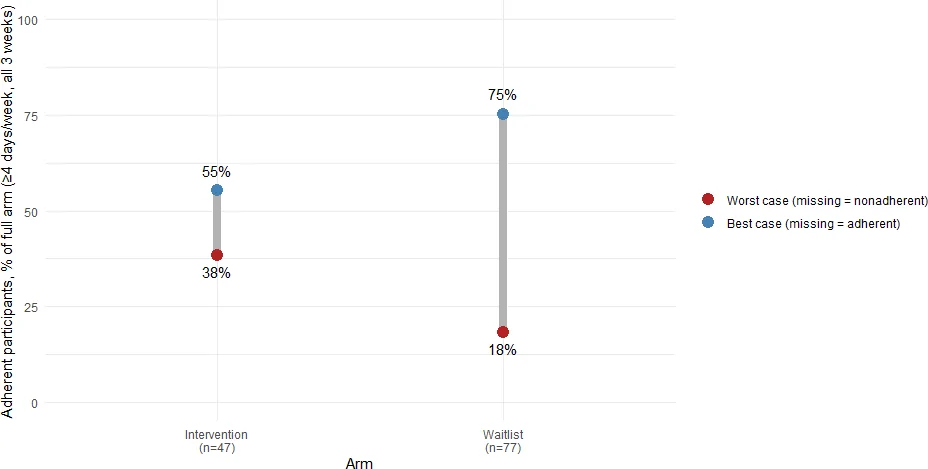
Adherence rate range under best-case and worst-case missing-data assumptions, by arm. Percentages reflect the proportion of the full arm (Intervention n=47; Waitlist n=77) meeting the ≥4 days/week, all-3-week adherence threshold. Worst case assumes all missing weeks were nonadherent; best case assumes all missing weeks were adherent. The wider range in the waitlist arm reflects a higher rate of missing adherence data rather than a difference in the underlying adherence rate.

**Figure 4.**
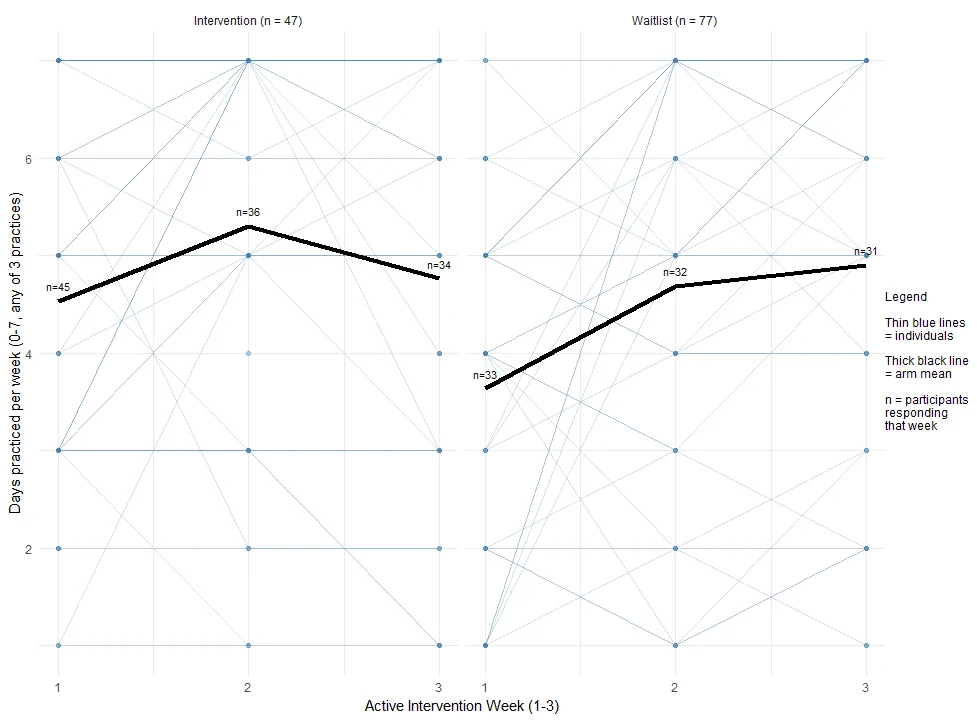
Individual and mean adherence trajectories during the 3-week active intervention period, by arm. Each thin blue line represents one participant’s weekly practice frequency, defined as the maximum days/week reported across the 3 practices (Isha Kriya, Nadi Shuddhi, and Simha Kriya); the thick black line shows the arm mean at each week. Because of the waitlist-controlled crossover design, “week” reflects each arm’s own active-intervention period rather than calendar time. Intervention arm weeks 1‐3 correspond to calendar weeks 1‐3, while for the waitlist arm, weeks 1‐3 correspond to calendar weeks 4‐6. Panel headers (N) show the total number of participants who reached the active intervention period in each arm while n=labels above the mean line indicate the number of participants who actually responded to the adherence survey at that specific week.

##### Acceptability

Acceptability was assessed qualitatively and is reported in the Qualitative Results, including participant accounts of the flexibility and accessibility of the digital format and their ability to sustain practice despite symptoms.

##### Secondary Outcomes

The LMM provided mean differences adjusted for age, gender, race, and ethnicity (Table 3). Significant group × time interactions indicated greater improvement in the intervention group across multiple POMS domains, including TMD (mean difference [MD]=−10, η²=.05; *P*=.02), positive mood (MD=2.9, η²=.05; *P*=.01), negative mood (MD=−7.5, η²=.04; *P*=.04), vigor (MD=1.7, η²=.03; *P*=.03), and fatigue (MD=−2.9, η²=.09; *P*=.001).

**Table 3.** Linear mixed effects model comparing changes in intervention group to waitlisted control from baseline to week 3 (postintervention). Linear mixed effects model adjusting for age, sex, race, ethnicity, and group x time interaction. Sample size (n=185) varies due to availability of outcome data at specific time points. Participants with baseline only were included in mixed-effects models but do not contribute to analyses requiring repeated measures.

Outcomes	Adjusted mean difference^a^ (95% CI)	*P* value	η^b^
PSS^c^	−0.53 (−2.7 to 1.6)	.63	0.002
POMS^d^			
TMD^e^	−10 (−19 to −1.6)	.02^f^	0.05
Positive	2.9 (0.66 to 5.1)	.01^f^	0.05
Vigor	1.7 (0.18 to 3.2)	.03^f^	0.04
Esteem-related affect	1.2 (0.09 to 2.3)	.03^f^	0.04
Negative	−7.5 (−15 to −0.44)	.04^f^	0.04
Tension	−1.6 (−3.6 to 0.48)	.13	0.02
Depression	−1.1 (−3.2 to 0.99)	.29	0.01
Anger	−1.1 (−2.6 to 0.44)	.16	0.02
Fatigue	−2.9 (−4.6 to −1.2)	.001^g^	0.09
Confusion	−0.80 (−2.3 to 0.67)	.28	0.01
SF-12^h^			
PCS12^i^	2.5 (−0.12 to 5.1)	.06	0.04
MCS12^j^	−0.60 (−4.4 to 3.2)	.75	0.009
MDP^k^			
Immediate perception domain	−5.5 (−9.4 to −1.6)	.007^l^	0.07
A1 breathing discomfort	−0.39 (−1.2 to 0.42)	.34	0.009
Sensory dimension	−5.1 (−8.5 to −1.7)	.003^l^	0.09
SSS-8^m^	−2.0 (−3.6 to −0.38)	.02	0.06

a Adjusted mean difference in change between intervention and waitlist control groups, estimated from the time × group interaction term in the linear mixed-effects model.

b η²>=.14 indicates a large effect size, η²>=.06 medium effect size, and η²>=.01 small effect size.

c PSS: Perceived Stress Scale.

d POMS: Profile of Mood States scale.

e TMD: total mood disturbance.

f *P*<.05.

g *P*<.001.

h SF-12: 12-Item Short Form Survey.

i PCS 12: physical component summary.

j MCS 12: mental component summary.

k MDP: Multidimensional Dyspnea Profile.

l *P*<.01.

m SSS-8: Somatic Symptom Scale-8.

The intervention group also showed significantly greater reductions in the MDP immediate perception domain (MD=−5.5, η²=.06; *P*=.007), MDP sensory dimension (MD=−5.1, η²=.09; *P*=.003), and SSS-8 scores (MD=−2.0, η²=.06; *P*=.02) compared with the waitlist group. There was no significant change in PSS (MD=−0.53, *η^2^*=0.002; *P*=.63), the MCS12 (MD=–0.60, *η^2^*=0.001; *P*=.75), and the PCS12 (MD=2.5, *η^2^*=0.04; *P*=.06) when comparing the intervention group to the waitlist group in the first 3 weeks of the study (Table 3). Three-week changes in median scores for statistically significant outcomes are illustrated in Figure 5.

**Figure 5.**
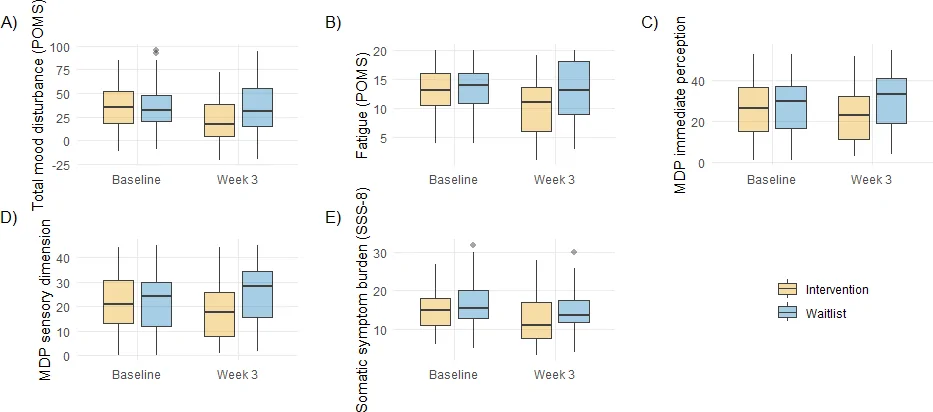
Baseline-to-week 3 changes in median scores for mood, dyspnea, and somatic symptom outcomes, by arm.(3A) Change in profile of mood states-total mood disturbance score. (3B) Change in profile of mood states-fatigue subscale score. (3C) Change in Multidimensional Dyspnea Profile - immediate perception subscale score. (3D) Change in Multidimensional Dyspnea Profile - sensory dimension subscale score. (3E) Change in somatic symptom scale – 8-item score. Boxes represent the interquartile range, the horizontal line is the median, whiskers are upper and lower adjacent values, and outliers are shown as individual points. MDP: Multidimensional Dyspnea Profile; POMS: Profile of Mood States; SSS-8: Somatic Symptom Scale-8.

To contextualize statistical versus clinical significance, between-group differences were compared with published minimal clinically important differences (MCIDs), noting all derive from populations with nonlong COVID. SF-12 MCIDs cluster around 3.3 (PCS) and 3.8‐5 (MCS) points, where observed differences (PCS +2.5, *P*=.06; MCS −0.6, *P*=.75) did not reach these thresholds. As SSS-8 MCIDs range from 3‐6 points, the observed reduction (−2; *P*=.02) was statistically significant but not clinically significant [41,42]. For the MDP perception domain, published MCIDs range from 3.02‐5.09 points across cardiorespiratory disease subgroups [43]. In our study, observed reductions (immediate perception: MD=−5.5; sensory qualities: MD=−5.1) exceeded this range. No MCID for the PSS or POMS was available in the literature.

### Harms

No adverse events, privacy breaches, or significant technical disruptions (eg, Zoom connectivity failures preventing participation) were reported during the study.

### Ancillary Analyses

Post hoc analyses were performed to further explore feasibility and efficacy of the meditation and breathing practices in a population with long COVID. The proportion meeting the adherence criterion depended on the threshold selected, ranging from 41.4% (24/58, ≥5 days/week) to 72.4% (42/58, ≥3 days/week), compared with 55.2% (32/58) under the primary definition (Table S2 in Multimedia Appendix 7). Baseline demographic and clinical characteristics were similar between retained and nonretained participants and between adherent and nonadherent participants (Tables S3 and S4 inMultimedia Appendix 7). Sensitivity analyses of the primary efficacy model showed similar findings: a pooled model combining both cohorts’ active-intervention periods (n=155) demonstrated a comparable overall effect with larger effect sizes than the primary model, and a per-protocol analysis showed no differences from the primary modified intent-to-treat (mITT) results (Tables S5 and S6 in Multimedia Appendix 7). Exploratory analyses examining adherence–outcome associations (considered secondary since adherence was determined postrandomization) showed no significant interactions with outcomes under complete-case, worst-case, or best-case missing-data assumptions, nor when adherence was modeled as a continuous gradient, except for a significant dose-response association with somatic symptom burden (*β*=−0.56, *P*=.015; Tables S7 and S8 in Multimedia Appendix 7). Full results and interpretation are provided in Multimedia Appendices 7 and 8.

### Qualitative Results

#### Overview

Thirty individual interviews (15 intervention and 15 waitlist) and 2 FGDs were analyzed to generate four themes relevant to the research question: (1) participants’ struggles with the medical system, (2) perceived benefits and challenges of digital medicine, (3) online support networks, and (4) the effect of the guided meditation and breathing practices (Isha Kriya, Nadi Shuddhi, and Simha Kriya). A visual map of these findings can be found in Multimedia Appendix 9.

#### Participants’ Struggles With the Medical System

Many participants expressed frustration with the medical system, stating that they felt unheard and that physicians were not up to date on long COVID treatment. One participant said:

Just acknowledge that it is real, that what the patient is feeling is real. And I understand there could be people out there, making it up or trying to ride the coattails of Long COVID, but there’s us who are legitimately sick

[Female, 47 years, Irish]

On lack of physician knowledge, an interviewee reported:

I think also very much felt like my primary care doctor and everyone else making it up on the fly, right? So, I felt like they were very caring but like no one really knew what to do

[Female, 67 years, Caucasian]

#### Perceived Benefits and Challenges of Digital Medicine

Participants shared their thoughts on the hybrid digital format of the intervention, which combined live Zoom sessions with prerecorded videos. This theme includes insights about the benefits and challenges from both individual interviews and an FGD. Many interviewees found the accessibility and flexibility of these formats helpful for incorporating the practices into their daily routines, despite their fatigue or difficulty attending in person. One participant noted:

The recorded videos were helpful—they gave me the flexibility to practice anytime during the day, without worrying about transportation or whether I had enough energy. That made it much easier to keep up with the practices, and I managed to do them once a day.

[Female, 52 years, Caucasian]

Some participants reported that brain fog made it difficult to follow the pace of some breathing practices despite appreciating the accessibility of the digital format. One participant explained:

Everyone said that the pace of that breathing was too fast for them. For me, I get distracted easily—I have a hard time paying attention, and it takes a lot of effort to stay focused. You need something simpler, as brain fog is a major problem among long COVID patients.

[Focus group, Female, 45 years, Caucasian]

Together, these findings illustrate that while the web-based format improved accessibility and flexibility, some participants experienced challenges performing the breathing practices because of cognitive symptoms such as brain fog.

#### Online Support Networks

In addition, participants discussed how in general, digital platforms became a substitute for in-person medical and social support while living with long COVID. They relied on telehealth, online peer groups, and virtual rehabilitation programs to navigate medical uncertainty, emotional isolation, and gaps in the health care system.

Digital medicine became both a bridge to connection and a tool for survival. One individual said:

One of the online groups I joined was this pulmonary rehabilitation group. Everything in New York had closed, so he started this online program. He would even work with us one-on-one, and that’s how I figured out who to see and what to do. I just did what he told me. Honestly, without that online support, I don’t know what I would’ve done

[Female, 62 years, Caucasian]

Another participant reported:

These Zoom programs helped me feel less alone and less scared. It’s sad that so many people are suffering, but it helped me feel less that I am not alone.

[Female, 45 years, Caucasian]

These digital networks not only reduced isolation but also helped participants learn about complementary approaches, such as our web-based guided meditation and breathing practices, that offered additional avenues for coping and self-management. As one participant mentioned:

I heard about your online practices through one of my online support groups, so I went online and signed up for it because I felt like I still had chest tightness

[Male, 33 years, Caucasian]

In this way, digital communities and digital medicine became essential resources for patients with long COVID actively searching for accessible treatment options.

#### The Effect of the Guided Meditation and Breathing Practices (Isha Kriya, Nadi Shuddhi, and Simha Kriya)

Participants reported improvements in symptoms, including feeling calmer and being able to breathe without fear, which are indicative of enhanced resilience. Resilience was noted as follows: (1) calming the mind using breath and awareness, and (2) rebuilding identity by recognizing oneself as a spiritual being. These abilities allowed individuals to navigate challenges with greater clarity and strength. A participant said:

I felt like I almost had a mental block about breathing because of all the breathing problems. I felt like I was almost scared to take a deep breath. Since doing these breathing exercises, I feel more comfortable, and even when I’m not actively doing them, there’s now a kind of mental trigger. Sometimes, during the day, I’ll take four or five really deep breaths. That helps keep me calm and reassures me that I can take a deep breath. There’s nothing physically wrong with me—I know I can do it.

[Female, 59 years, Caucasian]

On rebuilding identity, another participant stated:

You know, meditation makes me feel like I am a spiritual being. I have a soul I don’t have to be trapped in my mind, and I don’t have to worry about my body.

[Female, 61 years, Caucasian]

These reflections represent participants’ personal psychological and existential reframing of their relationship to their symptoms rather than a claim, taught or implied by the intervention, that long COVID lacks a physiological basis. Instructors were explicitly trained to affirm long COVID as a physiological condition throughout the program (see Methods, Intervention).

### Integrated Results

Integration of quantitative and qualitative findings revealed clear convergence, divergence, and expansion, offering a multidimensional understanding of the intervention’s effects (Figure 6). The research team observed convergence among the quantitative and qualitative data in areas where qualitative data showed improved breathing confidence and calmness and clarified why participants felt calmer and more in control of their bodies. This aligned with quantitative improvements in dyspnea, mood disturbance, and somatic symptom burden. As noted above, the clinical significance of these improvements varied: the dyspnea improvement exceeded published benchmarks for clinical relevance, while the somatic symptom improvement, though statistically significant, was more modest in magnitude.

**Figure 6.**
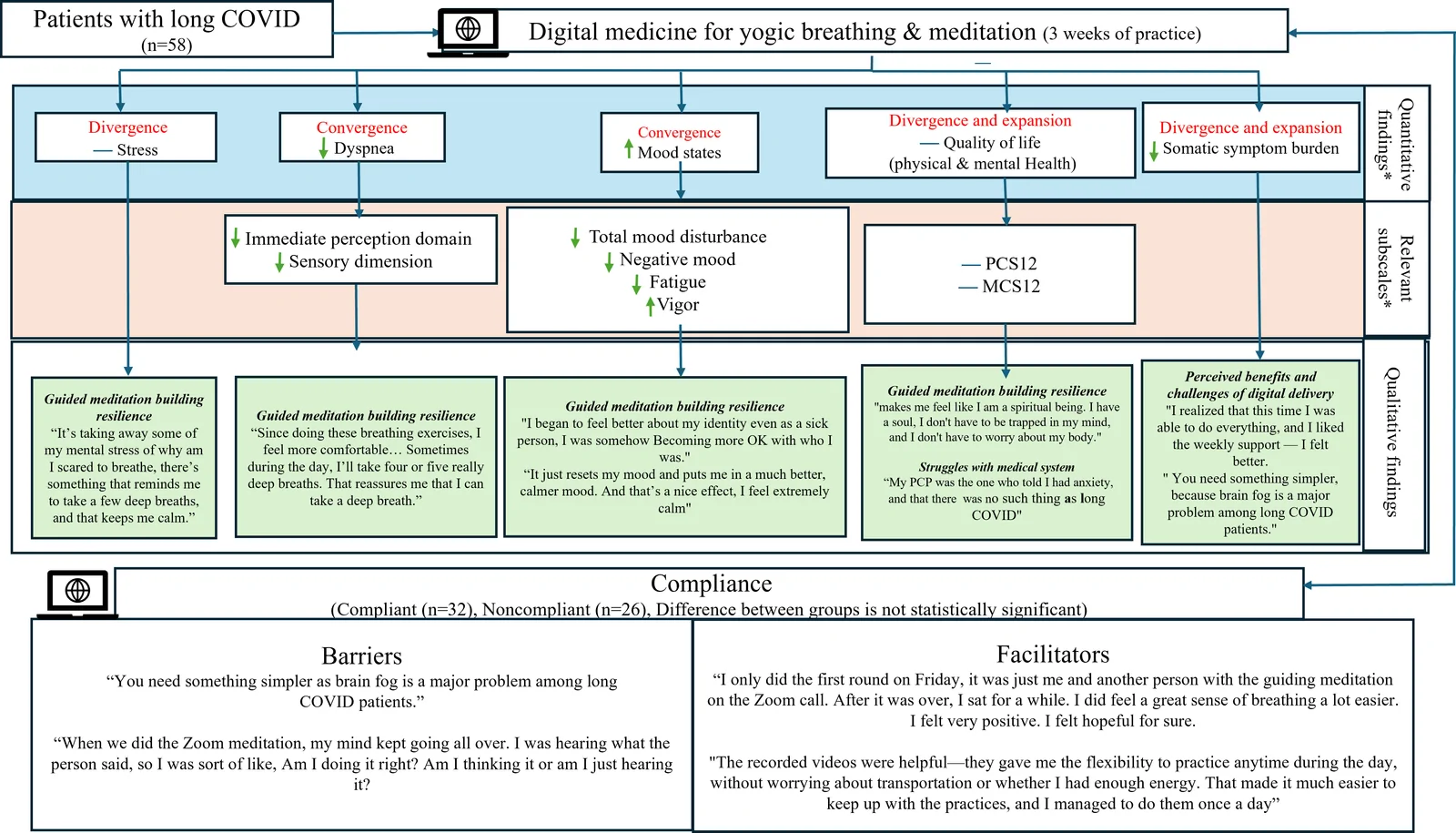
Joint display of quantitative and qualitative findings exploring the feasibility and preliminary efficacy of digitally delivered guided breathing and meditation for long COVID symptoms. Blue dashes represent no change. Green arrows represent statistically significant positive improvements in symptoms according to quantitative findings. The (n=58) represents the number of participants who had complete 3-week adherence data. Interview quotes from qualitative findings have been included to demonstrate convergence, divergence, and expansion from the integration of quantitative and qualitative findings*.* *Linear mixed effects models adjusted for age, gender, race, ethnicity, and group x time interaction. Please refer to Table 3 for details. MCS 12: mental component summary; PCS 12: physical component summary.

Divergence was observed between the quantitative and qualitative findings for perceived stress and quality of life. Although the PSS and both SF-12 components (physical and mental) did not demonstrate statistically significant improvement, participants described reduced stress, greater emotional calmness, and improved coping during the qualitative interviews. Many participants also described ongoing struggles with the medical system and brain fog, which may have limited measurable improvement on standardized stress and quality-of-life scales even when subjective experiences were positive. No qualitative themes directly contradicted the quantitative findings. Instead, the observed divergence reflected differences in emphasis between the quantitative measures and participants’ lived experiences, providing complementary insights into the intervention’s effects.

Expansion occurred through themes not captured by quantitative scales: digital delivery, medical-system experience, social connection, and identity. Participants valued the digital format’s flexibility, reduced physical burden, and ability to accommodate fatigue, though cognitive symptoms such as brain fog made it difficult to follow the pace of some practices. Many also described feeling unheard by physicians and encountering inconsistent guidance for managing long COVID, a structural and relational dimension of their illness experience that helps explain their motivation for seeking a self-directed, complementary program such as this one. Qualitative data further revealed social connection through online peer groups and virtual rehabilitation spaces, which helped participants navigate isolation and find trusted guidance, alongside psychosocial and existential insights, including a sense of rebuilding identity beyond their illness, that extend beyond the measured outcomes.

## Discussion

### Principal Findings

Taken together, the study findings suggest that this web-based yogic breathing and meditation program is feasible for supporting symptom management in people with long COVID in terms of demand, adherence, and acceptability, while retention remains a key challenge for future implementations. The program drew over 700 referrals and sign-ups, indicating strong recruitment demand, and secondary analyses suggested a promising preliminary signal favoring the intervention across mood, dyspnea perception, and somatic symptom burden. Only the breathing-related improvement exceeded thresholds considered clinically meaningful in other populations. Though statistically significant, the somatic symptom improvement was more modest and no comparable benchmark exists for mood. These comparisons should be interpreted cautiously, as clinical significance of changes in individuals with long COVID remains uncertain.

Integration of quantitative and qualitative findings (Results, Integrated Results; Figure 6) showed convergence for dyspnea, mood, and somatic symptom burden, and divergence for stress and quality of life, where subjective improvement was not mirrored by significant quantitative change. Rather than reflecting contradiction, this divergence underscores the value of the mixed-methods design in surfacing improvements (eg, rebuilding identity and social connection through digital platforms) that standardized instruments were not designed to detect, while also reflecting stressors beyond the intervention’s reach. Qualitative interviews also showed that digital delivery played a central role in shaping feasibility. The program’s blended synchronous (live Zoom) and asynchronous (recorded video) model let participants engage in real time or on their own schedule, balancing live guidance with the flexibility to accommodate fluctuating symptom burden. Participants valued the accessibility of remote delivery, which allowed them to learn the practices despite fatigue and challenges attending in-person sessions. At the same time, brain fog highlighted the importance of pacing and reducing instructional complexity, considerations relevant to long COVID-specific intervention design regardless of delivery format. Additionally, since delivery was entirely remote, the program has potential for scalable delivery without the travel and facility requirements of in-person MBIs.

Adherence status was not significantly associated with change in any secondary outcome, whether modeled as a binary threshold under complete-case, worst-case, or best-case missing-data assumptions, or as a continuous gradient of practice frequency, which showed directionally favorable but largely nonsignificant associations across outcomes. Because adherence is determined after randomization, these associations cannot be interpreted causally. Interestingly, baseline symptom burden did not differ between adherent and nonadherent participants, suggesting that initial symptom severity was unlikely to be a major determinant of adherence. Although this reduces concern that baseline severity confounded the exploratory adherence–outcome associations, it does not exclude the possibility that symptom changes during the intervention influenced participant engagement. Additional research is needed to determine whether greater practice exposure leads to improved outcomes and to better characterize potential dose-response relationships.

### Comparison With Prior Work

The study findings are consistent with the growing literature on digital rehabilitation approaches for individuals with long COVID. A scoping review of digital interventions incorporating psychological and physical components, including mindfulness and breathing exercises, found improvements in physiological health, reductions in breathlessness and fatigue, and enhanced ability to sustain daily activities [44]. Although most interventions lasted 7 weeks to 2 months, these findings were comparable to the improvements observed following the 3-week Breathing and Wellness program, suggesting that even brief digital MBIs may provide meaningful symptom support.

The absence of significant changes in PSS and SF-12 scores despite qualitative reports of reduced stress may partly reflect the broad, noncondition-specific nature of these measures, which may have limited their ability to capture the aspects of change described by participants. Longitudinal research suggests that a substantial proportion of PSS variance may reflect stable, trait-like characteristics rather than short-term fluctuations, particularly over brief assessment windows [45]. Future studies incorporating longer follow-up periods and long COVID-specific stress measures may help clarify whether persistent perceived stress reflects the chronic nature of the condition, limitations in measurement sensitivity, or both.

Findings from other MBIs in post–COVID-19 condition further contextualize the present results. A recent pilot RCT of a yoga program for post–COVID-19 condition (Breathe Easy) offers a useful comparison to this study [46]. Although both interventions incorporated alternate-nostril breathing, Breathe Easy emphasized physical postures over seated breathing and meditation, used a hybrid delivery model (initially in-person with later virtual options), had a longer intervention period (12 vs 3 weeks), and enrolled a smaller, predominantly female sample (n=29 vs 58) using the SF-36 rather than the SF-12. Despite these differences, the studies converged on 2 important implementation findings: participants valued virtual access when fatigue, pain, or transportation barriers limited in-person attendance, and instructors incorporated pacing strategies in response to symptom exacerbation. Taken together, these findings suggest that flexibility in delivery and pacing may be useful considerations when adapting MBIs for people with long COVID.

In alignment with the improvements observed in secondary outcomes, systematic reviews and meta-analyses on yoga and meditation for similar conditions like asthma, chronic fatigue, and chronic respiratory diseases show improvement in dyspnea, pulmonary function, and quality of life [47,48]. The specific practices included in the Breathing and Wellness program have demonstrated benefits in prior research as well. Simha Kriya was associated with a reduction in COVID-19–related distress among health care workers after 4 weeks [23]. Nadi Shuddhi was associated with reduced stress and improved well-being among undergraduate students during the COVID-19 pandemic [22]. Isha Kriya was associated with both immediate and short-term benefits: a single session reduced mood disturbance among health care workers, while 2 weeks of regular practice improved self-reported anxiety and depression symptoms [15,20]. Although the effects observed here cannot be attributed to any single practice in isolation, the combined 3-week program was associated with improvements in breathing and mood states in a direction broadly consistent with those reported in prior research. Enhanced autonomic regulation, reduced sympathetic arousal, and improved mind–body integration are commonly proposed mechanisms in yoga- and meditation-based therapies and may plausibly contribute to the improvements observed. However, this study did not include physiological measures and cannot speak to mechanism directly [17,18].

### Strengths and Limitations

A key strength of this study is its mixed-methods design, which enabled quantitative outcomes to be interpreted alongside participants’ lived experiences, providing insight into how and why the intervention influenced mood, dyspnea, and somatic symptoms beyond what standardized measures alone can capture. The study also characterized adherence in detail by distinguishing withdrawal from nonresponse and using best- and worst-case bounds to describe uncertainty rather than relying on potentially unstable imputation. This transparent approach provides a more complete representation of participant engagement.

Several limitations should be considered. Concurrent treatments were not assessed, limiting characterization of participants’ broader care experiences. However, given 1:1 randomization, concurrent care would be expected to be approximately balanced across groups [49]. Future trials should collect these data to enable adjustment and subgroup analyses. Outcomes were self-reported, and participants could not be blinded to group assignment, making expectancy effects and the supportive group environment potential contributors to observed improvements. These limitations are common in behavioral interventions and are difficult to fully separate from intervention effects [50]. Additionally, most outcome measures were generic instruments developed and validated in other populations, whereas long COVID-specific formal psychometric validation remains limited [33-35,37,39]. Objective physical performance measures were not included due to concerns that additional assessments would compound participant burden and attrition given existing long COVID symptom burden. Future studies should incorporate objective measures and longer follow-up to complement self-reported outcomes and determine the durability of observed improvements.

Another limitation was that participants were predominantly non-Hispanic White women, limiting conclusions about intervention acceptability and effectiveness across more diverse populations. Although models adjusted for race and gender and findings remained consistent, this sample composition may reflect both the demographic distribution of individuals affected by long COVID and disparities in diagnosis, referral, and research participation [1]. Given evidence that ethnic minority populations remain underrepresented in long COVID research despite potentially greater burden of disease, future studies should prioritize purposive recruitment of underserved communities [51]. The requirement for PCR-confirmed SARS-CoV-2 infection may also limit generalizability, as neither the WHO nor the subsequent NASEM definition of long COVID requires laboratory confirmation of the initial infection [6,30]. This means that individuals without access to testing or those with asymptomatic infections may therefore have been excluded despite having long COVID, particularly given the limited availability of testing early in the pandemic [6]. The study sample may consequently underrepresent individuals with unconfirmed infections and may not fully reflect the broader population with long COVID.

Finally, retention was challenging, consistent with the fluctuating and persistent nature of long COVID symptoms [7]. As with many eHealth interventions, engagement may also be influenced by factors beyond intervention efficacy, including participant motivation, personal values, and familiarity with digital technologies [52,53]. Participants who consented to participate may have been more motivated, comfortable with digital platforms, or interested in mind-body approaches, which may limit generalizability. Reduced engagement with the intervention may similarly reflect symptom burden, technology-related barriers, or other contextual factors rather than lack of acceptability [52]. Attrition appeared more related to the burden of study participation (particularly repeated weekly assessments) than to the intervention itself. Retention and engagement challenges have similarly been reported in other long COVID intervention trials [54-56]. Attrition patterns differed by study arm: intervention-arm attrition reflected both withdrawal and nonresponse, whereas waitlist-arm attrition was primarily due to participants remaining enrolled but discontinuing surveys before receiving the intervention. Because these individuals were unlikely to complete qualitative interviews, their experiences are underrepresented, limiting our ability to determine the reasons for disengagement. Nonetheless, the pattern highlights the importance of minimizing assessment burden in delayed-start designs. Future studies should consider shorter surveys, passive or streamlined symptom tracking, and more active retention strategies such as phone or video check-ins.

### Conclusions

This study demonstrates the feasibility and potential benefit of a brief, fully web-based yogic breathing and meditation intervention adapted specifically for long COVID. By combining Nadhi Shuddhi, Simha Kriya, and Isha Kriya practices with a mixed-methods approach, this trial extends prior digital rehabilitation research by capturing both clinical outcomes and lived experiences of recovery, including changes in mood, dyspnea, agency, and coping in the context of MBI practices. The low-burden, scalable delivery model may offer a practical complementary approach for individuals facing barriers to in-person care. Future studies should evaluate longer-term effects, confirm dose-response relationships, optimize digital delivery for cognitive symptoms such as brain fog, and examine integration into broader telehealth and rehabilitation pathways.

## Supplementary material

10.2196/89631 Multimedia Appendix 1 Trial protocol.

10.2196/89631 Multimedia Appendix 2 Meditation and breathing intervention practice links.

10.2196/89631 Multimedia Appendix 3 Expanded intervention and instructor training description.

10.2196/89631 Multimedia Appendix 4 Compliance questionnaire.

10.2196/89631 Multimedia Appendix 5 Somatic symptom scale 8-item questionnaire.

10.2196/89631 Multimedia Appendix 6 Qualitative interview questions.

10.2196/89631 Multimedia Appendix 7 Ancillary analysis tables.

10.2196/89631 Multimedia Appendix 8 Ancillary analyses extended results and interpretation.

10.2196/89631 Multimedia Appendix 9 Thematic summary of qualitative findings from a web-based meditation and breathwork program for patients with long COVID derived using conventional content analysis.

10.2196/89631 Checklist 1 CONSORT-*eHEALTH* checklist (V 1.6.1).

10.2196/89631 Checklist 2 CONSORT checklist.

## Abbreviations

BIDMC: Beth Israel Deaconess Medical Center
CONSORT: Consolidated Standards of Reporting Trials
CONSORT E-HEALTH: Consolidated Standards of Reporting Trials of Electronic and Mobile Health Applications and Online Telehealth
COREQ: Consolidated Criteria for Reporting Qualitative Research
FGD: focus group discussion
GRAMMS: Good Reporting of a Mixed Methods Study
HIPAA: Health Insurance Portability and Accountability Act
IRB: institutional review board
LMM: linear mixed-effects model
MBI: mind-body intervention
MCAR: missing completely at random
MCID: minimal clinically important difference
MCS: mental component summary
MD: mean difference
MDP: Multidimensional Dyspnea Profile
mITT: modified intent-to-treat
NASEM: National Academies of Sciences, Engineering, and Medicine
PCR: polymerase chain reaction
PCS: physical component summary
POMS: Profile of Mood States
PSS: Perceived Stress Scale
RCT: randomized controlled trial
SF-12: 12-Item Short Form Survey
SSS-8: Somatic Symptom – 8 Item
TMD: total mood disturbance
WHO: World Health Organization
