# Supplementary material for: Web-Based Isha Kriya Meditation and Breathwork in Long COVID: Mixed Methods Randomized Controlled Pilot Study
Source: J Med Internet Res. 2026 Nov 12;28:e89631. doi: 10.2196/89631 (PMC13758968; doi:10.2196/89631)
Supplement: Supplementary file 1 [file jmir-v28-e89631-s005.pdf]

ICMC

International Center for
Multimorbidity and
Complexity in Medicine
www.multimorbidity.org

Somatic Symptom Scale-8 (SSC-8)

English Version

Gierk B, Kohlmann S, Kroenke K, Spangenberg L, Zenger M, Brähler E, & Löwe B. (2014). The Somatic Symptom Scale–8 (SSS-8): A brief measure of somatic symptom burden. JAMA Internal Medicine, 174(3), 399–407.

Patient ID: _______________ Date: ______________

Notes:

Patient ID: _____________ Date: ____________

Somatic Symptom Scale 8 (SSS-8)

During the <u>past 7 days</u>, how much have you been bothered by any of the following problems?	**Not at all**	**A little bit**	**Somewhat**	**Quite a bit**	**Very much**
1. Stomach or bowel problems	0	1	2	3	4
2. Back pain	0	1	2	3	4
3. Pain in your arms, legs, or joints	0	1	2	3	4
4. Headaches	0	1	2	3	4
5. Chest pain or shortness of breath	0	1	2	3	4
6. Dizziness	0	1	2	3	4
7. Feeling tired or having low energy	0	1	2	3	4
8. Trouble sleeping	0	1	2	3	4

Column totals: _____ + _____ + _____ + _____ + _____

Total score: ______________

Gierk B, Kohlmann S, Kroenke K, Spangenberg L, Zenger M, Brähler E, & Löwe B. (2014). The Somatic Symptom Scale–8 (SSS-8): A brief measure of somatic symptom burden. JAMA Internal Medicine, 174(3), 399–407.

Somatic Symptom Scale 8 (SSS-8)

Description 4
Structure and Administration 4
Scoring and Interpretation 5
Recommended Cut-off Scores 5
Psychometric Properties 6
Key Research Findings 6
Clinical Utility & Limitations 7
Advantages 7
Limitations 7
References 8

Description

The **Somatic Symptom Scale-8 (SSS-8)** is a brief, validated self-report instrument designed to assess the severity of somatic symptom burden. It serves as a short form of the Patient Health Questionnaire-15 (PHQ-15) and aligns with the diagnostic framework of **Somatic Symptom Disorder (SSD)** as defined by the DSM-5. The SSS-8 enables efficient screening of patients in primary care, mental health, and psychosomatic settings, identifying individuals with potentially disabling physical symptoms, regardless of whether a clear medical explanation exists (Gierk et al., 2014).

The scale captures the patient's subjective experience of somatic symptoms over the previous seven days. Its simplicity makes it a useful tool for both clinical practice and research, facilitating early detection and guiding further diagnostic evaluations.

Structure and Administration

The SSS-8 comprises **eight items**, each representing a common somatic symptom. These items were selected based on their prevalence in clinical populations and their contribution to symptom burden and healthcare utilization.

Each of the eight items is scored on a **five-point Likert scale**, assessing how much the respondent was bothered by each symptom over the **past seven days**:

Response Option	Score
Not at all	0
A little bit	1
Somewhat	2
Quite a bit	3
Very much	4

The questionnaire is **self-administered**, but it can also be completed through **clinician interviews** or **computerized assessments**. It typically takes **1-2 minutes** to complete.

Scoring and Interpretation

Total SSS-8 scores range from **0 to 32**. Higher total scores reflect greater severity of somatic symptoms and are associated with increased functional impairment and healthcare use (Gierk et al., 2014).

SSS-8 Total Score	Severity Level	Clinical Interpretation
0-3	Minimal somatic symptom burden	No clinical intervention typically required
4-7	Low symptom burden	Monitor and provide psychoeducation.
8-11	Medium symptom burden	**Further assessment recommended.** Evaluate for functional impairment and possible co-occurring psychological factors (e.g., anxiety, depression). Consider referral for psychoeducation, behavioral interventions, or low-intensity psychotherapy.
12-15	High symptom burden	**Comprehensive diagnostic assessment needed.** Screen for Somatic Symptom Disorder (SSD) and other mental health conditions. Multidisciplinary intervention may be appropriate, including psychological treatment (e.g., Cognitive Behavioral Therapy) and coordination with primary care.
16-32	Very high symptom burden	**Immediate and integrated care recommended.** High likelihood of significant functional impairment and comorbid mental health conditions. A thorough psychosomatic evaluation is advised. Multimodal treatment, involving mental health specialists, primary care, and possibly physical therapy, should be considered.

These interpretations should always be adapted to the **clinical context**, including comorbid medical conditions and patient preferences. Patients with **scores ≥12** often benefit from a **biopsychosocial approach**, addressing both physical and psychological components.

Recommended Cut-off Scores

Recommended thresholds vary depending on the clinical context and target population:

- **≥8 points**: Indicates **clinically relevant somatic symptom burden**.
- **≥12 points**: Suggests **high symptom burden** often associated with **functional impairment**, **anxiety**, and **depression**.
- **≥16 points**: Reflects **very high somatic symptom burden**, frequently requiring comprehensive psychosomatic assessment and intervention (Gierk et al., 2014).

Cut-off scores may need adjustment for different cultural populations or settings to optimize sensitivity and specificity (Li et al., 2022; Cao et al., 2022).

Psychometric Properties

The Somatic Symptom Scale-8 (SSS-8) demonstrates strong psychometric properties across diverse populations and clinical settings. It has been validated in multiple languages, including German, Chinese, Greek, Russian, Korean, and Japanese, confirming its international applicability (Pollo et al., 2022; Petrelis & Domeyer, 2021; Li et al., 2022; Zolotareva, 2022).

Metric	Value	Reference
**Sensitivity (cut-off ≥12)**	**0.72** (72%)	Toussaint et al., 2019
**Specificity (cut-off ≥12)**	**0.59** (59%)	Toussaint et al., 2019
**Positive Predictive Value (PPV)**	**0.74** (74%) (depending on population)	Toussaint et al., 2019
**Negative Predictive Value (NPV)**	**0.64** (64%)	Toussaint et al., 2019
**Sensitivity (cut-off ≥9, Chinese sample)**	**0.80** (80%)	Cao et al., 2022
**Specificity (cut-off ≥9, Chinese sample)**	**0.67** (67%)	Cao et al., 2022
**Area Under the Curve (AUC)**	**0.73** (SSS-8 alone); 0.84 (with SSD-12)	Cao et al., 2022
**Internal Consistency (Cronbach's Alpha)**	**0.81-0.85** (good to excellent)	Gierk et al., 2014; Zolotareva, 2022
**Test-Retest Reliability (ICC)**	**> 0.99** (excellent stability)	Petrelis & Domeyer, 2021
**Construct Validity**	Strong correlations with PHQ-15, PHQ-9, GAD-7, SSD-12	Toussaint et al., 2019; Li et al., 2022

Key Research Findings

- The SSS-8 is as effective as longer somatic symptom questionnaires (e.g., PHQ-15) while being substantially shorter and easier to administer (Gierk et al., 2014).
- Higher SSS-8 scores are significantly associated with functional impairment, health anxiety, and comorbid psychiatric conditions such as depression and generalized anxiety disorder (Hüsing et al., 2018).
- Cross-cultural validations demonstrate robust psychometric performance across various populations, including translations into German, Greek, Chinese, Russian, Korean, and Japanese (Pollo et al., 2022; Li et al., 2022; Zolotareva, 2022).
- Combining the SSS-8 with the SSD-12 improves diagnostic accuracy for DSM-5 Somatic Symptom Disorder (Toussaint et al., 2019).

Clinical Utility & Limitations

Advantages

- **Brief and user-friendly**: Requires only 1-2 minutes to complete, making it feasible for routine use in primary care and psychiatric settings.
- **Validated and reliable**: Demonstrates strong psychometric properties across diverse settings.
- **Culturally adaptable**: Validated translations exist for use in different languages and cultural contexts.
- **Useful for screening and monitoring**: Sensitive to changes over time, aiding in treatment monitoring (Pollo et al., 2022).

Limitations

- **Lack of diagnostic specificity**: Does not differentiate between medically explained and unexplained symptoms.
- **Risk of over-identification**: Higher scores can occur in individuals with chronic medical illnesses unrelated to SSD.
- **Limited scope**: Does not assess the cognitive or behavioral features necessary for a formal DSM-5 diagnosis of SSD (Toussaint et al., 2019).
- **Cultural considerations**: Cut-off thresholds may need adjustment based on population-specific factors (Li et al., 2022).

References

1. **Cao J, Wei J, Fritzsche K, Toussaint AC, Li T, Zhang L, Zhang Y, Chen H, Wu H, Ma X, Li W, Ren J, Lu W, Leonhart R.** Detecting DSM-5 somatic symptom disorder in general hospitals in China: B-criteria instrument has better accuracy-A secondary analysis. Front Psychiatry. 2022 Oct 20;13:935597. doi: 10.3389/fpsyt.2022.935597. PMID: 36339843; PMCID: PMC9634742.

2. **Gierk B, Kohlmann S, Kroenke K, Spangenberg L, Zenger M, Brähler E, Löwe B.** The somatic symptom scale-8 (SSS-8): a brief measure of somatic symptom burden. JAMA Intern Med. 2014 Mar;174(3):399-407. doi: 10.1001/jamainternmed.2013.12179. PMID: 24276929.

3. **Pollo CF, Meneguin S, Miot HA, de Oliveira C.** Translation, cultural adaptation and validation of the Somatic Symptom Scale-8 (SSS-8) for the Brazilian Portuguese language. BMC Prim Care. 2022 Sep 5;23(1):222. doi: 10.1186/s12875-022-01836-2. PMID: 36064343; PMCID: PMC9442982.

4. **Li T, Wei J, Fritzsche K, Toussaint AC, Zhang L, Zhang Y, Chen H, Wu H, Ma X, Li W, Ren J, Lu W, Leonhart R.** Validation of the Chinese version of the Somatic Symptom Scale-8 in patients from tertiary hospitals in China. Front Psychiatry. 2022 Sep 28;13:940206. doi: 10.3389/fpsyt.2022.940206. PMID: 36276338; PMCID: PMC9583900.

5. **Petrelis M, Domeyer PR.** Translation and validation of the Greek version of the Somatic Symptom Scale-8 (SSS-8) in patients with chronic low back pain. Disabil Rehabil. 2022 Aug;44(16):4467-4473. doi: 10.1080/09638288.2021.1900415. Epub 2021 Mar 16. PMID: 33725461.

6. **Toussaint A, Hüsing P, Kohlmann S, Löwe B.** Detecting DSM-5 somatic symptom disorder: criterion validity of the Patient Health Questionnaire-15 (PHQ-15) and the Somatic Symptom Scale-8 (SSS-8) in combination with the Somatic Symptom Disorder - B Criteria Scale (SSD-12). Psychol Med. 2020 Jan;50(2):324-333. doi: 10.1017/S003329171900014X. Epub 2019 Feb 7. PMID: 30729902.

7. **Hüsing P, Löwe B, Toussaint A.** Comparing the diagnostic concepts of ICD-10 somatoform disorders and DSM-5 somatic symptom disorders in patients from a psychosomatic outpatient clinic. J Psychosom Res. 2018 Oct;113:74-80. doi: 10.1016/j.jpsychores.2018.08.001. Epub 2018 Aug 2. PMID: 30190052.

8. **Zolotareva A.A.** Factor Structure of the Russian Version of the Somatic Symptom Scale-8 (SSS-8). *Konsul'tativnaya psikhologiya i psikhoterapiya = Counseling Psychology and Psychotherapy,* 2022. Vol. 30, no. 3, pp. 8–20. DOI: 10.17759/cpp.2022300302. (In Russ., abstr. in Engl.)
