# Supplementary material for: Web-Based Isha Kriya Meditation and Breathwork in Long COVID: Mixed Methods Randomized Controlled Pilot Study
Source: J Med Internet Res. 2026 Nov 12;28:e89631. doi: 10.2196/89631 (PMC13758968; doi:10.2196/89631)
Supplement: Supplementary file 2 [file jmir-v28-e89631-s009.pdf]

Experiences of People With Long COVID

Experiences with Long COVID and a Web-Based Isha Kriya Meditation and Breathwork Practices

Theme 1. ***Participants' struggles with the medical system***

Feeling unheard, limited understanding, and difficulty accessing appropriate care

- Feeling unheard and needing symptoms to be acknowledged
- Limited physician knowledge about Long COVID
- Uncertainty about treatment and management
- Frustration with the medical system

"I think it also very much felt like my primary care doctor
and everyone else making it up on the fly, right?
So, I felt like they were very caring, but like no one really knew what to do

Theme 2. ***Perceived benefits and challenges of digital medicine***

Accessible and flexible, but with real limitations

Perceived benefits

- Accessibility and flexibility
- Ability to practice at convenient times
- Reduced transportation burden
- Ability to participate despite fatigue or low energy

Perceived challenges

- Difficulty with pacing
- Difficulty maintaining attention
- Brain fog and concentration difficulties

"The recorded videos were helpful—they gave me the flexibility to
practice anytime during the day,
without worrying about transportation or whether I had enough energy.
That made it much easier to keep up with the practices, and I managed
to do them once a day."

Theme 3. Online Support Networks

Connection, shared understanding, and practical guidance

- Connection with others experiencing Long COVID
- Reduced feelings of isolation
- Online peer and rehabilitation support
- Guidance for navigating illness and care
- Discovery of complementary approaches

"These Zoom programs helped me feel less alone
and less scared.
It's sad that so many people are suffering,
but it helped me feel less that I am not alone."

Theme 4. ***The effect of the guided meditation and breathing practices (Isha Kriya, Nadi Shuddhi, and Simha Kriya)***

Calming the mind, greater breathing confidence and a renewed sense of self

- Calming through breath and awareness
- Greater confidence with breathing
- Emotional regulation
- Rebuilding identity and self-perception

"Sometimes, during the day, I'll take four or five really deep breaths.
That helps keep me calm and reassures me that I can take a deep breath.
There's nothing physically wrong with me—I know I can do it"
