# Supplementary material for: Web-Based Isha Kriya Meditation and Breathwork in Long COVID: Mixed Methods Randomized Controlled Pilot Study
Source: J Med Internet Res. 2026 Nov 12;28:e89631. doi: 10.2196/89631 (PMC13758968; doi:10.2196/89631)
Supplement: Supplementary file 5 [file jmir-v28-e89631-s001.docx]

PART B

**STUDY DESCRIPTION**

| **Title of Protocol** | **Yogic Breathing and Guided Meditation for Long Covid Symptoms** |
| --- | --- |
| **Short Title** | **Covid No Longer** |
| Principal Investigator | **Balachundhar Subramaniam, MD, MPH** |

# B1. PURPOSE OF PROTOCOL

This study aims to assess the impact of brief digitally delivered breathing practice and guided meditation on post-Covid physical and mental symptoms in Long Covid Patients.

Specific aims include:

**1a**.To assess the level of compliance in those learning the online intervention over a period of 3 weeks, compared to waitlisted control.

**1b**.To quantitatively assess the impact of the intervention on participants' stress levels using a validated questionnaire over a period of 3 weeks, compared to waitlisted control.

**2.**To quantitatively assess the impact of the intervention on mood state, somatic symptoms, breathing discomfort and quality of life using validated scales over a period of 3 weeks, compared to waitlisted control.

**3-** To qualitatively explore participants’ perspectives on their long COVID symptoms, physician care, and impact of interventions on their symptoms.

#### B2. SIGNIFICANCE AND BACKGROUND FOR THE STUDY

It is estimated that about 10% of those who are infected by Covid 19 become long haulers (Long Covid condition)^1^. Other than its damaging effect on the lungs, Covid 19 infection can lead to inflammation and damage in other organs including heart and brain^2^, multisystem inflammatory syndrome (MIS) and autoimmune conditions^3^ which may contribute to Long Covid symptoms. According to a recent study following over 200k patients within 6 months after Covid infection, over 33% were diagnosed with a neurological or psychiatric condition with 12.8% receiving their first such diagnosis. Anxiety disorders had the highest prevalence among these conditions^4^.

Long Covid symptoms can last weeks to months after the initial Covid infection or can appear weeks after. It can occur in those with mild disease or asymptomatic patients. Most commonly reported symptoms of Long Covid are^3^:

- Tiredness or fatigue
- Difficulty thinking or concentrating (sometimes referred to as “brain fog”)
- Headache
- Loss of smell or taste
- Dizziness on standing
- Fast-beating or pounding heart (also known as heart palpitations)
- Chest pain
- Difficulty breathing or shortness of breath
- Cough
- Joint or muscle pain
- Depression or anxiety
- Fever
- Symptoms that get worse after physical or mental activities

To this date, nearly 180 million people have been infected with Covid19 and over 3 million have lost their lives worldwide^5^. The increasing prevalence of patients with Long Covid symptoms and the lack of effective solutions to address their condition, creates an urgent need for non-pharmacological interventions that are effective and scalable and can be delivered online to accommodate for the limitations due to the Covid pandemic.

Yogic Breathing and Meditation techniques have been shown to have various health benefits including improving pulmonary function and mental health^6–8^. Prominent health institutions are recommending breathing exercise to their Covid patients to assist their respiratory recovery^9^. Meditation and breathing is also shown to help with Covid related stress and anxiety^10,11^.

The purpose of this study is to investigate the impact of a set of **simple, safe and scalable, digitally delivered Yogic Breathing and Guided Meditation practices** on the neuropsychological, breathing discomfort, somatic symptoms and quality of life in Long Covid patients over a period of 3 weeks. The intervention consists of two Yogic Breathing practices (Simha Kriya and Nadi Shuddhi) and a guided meditation (Isha Kriya).

Our recent studies using these interventions have shown feasibility and acceptance as well as Improved perceived stress in healthy subjects^8,11,12^.

**References:**

1.	Greenhalgh T, Knight M, A’Court C, Buxton M, Husain L. Management of post-acute covid-19 in primary care. *BMJ*. 2020;370:m3026. doi:10.1136/bmj.m3026

2.	COVID-19 (coronavirus): Long-term effects. Mayo Clinic. Accessed May 3, 2021. https://www.mayoclinic.org/diseases-conditions/coronavirus/in-depth/coronavirus-long-term-effects/art-20490351

3.	CDC. COVID-19 and Your Health. Centers for Disease Control and Prevention. Published February 11, 2020. Accessed May 21, 2021. https://www.cdc.gov/coronavirus/2019-ncov/long-term-effects.html

4.	Taquet M, Geddes JR, Husain M, Luciano S, Harrison PJ. 6-month neurological and psychiatric outcomes in 236 379 survivors of COVID-19: a retrospective cohort study using electronic health records. *Lancet Psychiatry*. 2021;8(5):416-427. doi:10.1016/S2215-0366(21)00084-5

5.	WHO Coronavirus (COVID-19) Dashboard. Accessed June 27, 2021. https://covid19.who.int

6.	Abel AN, Lloyd LK, Williams JS. The effects of regular yoga practice on pulmonary function in healthy individuals: a literature review. *J Altern Complement Med N Y N*. 2013;19(3):185-190. doi:10.1089/acm.2011.0516

7.	Budhi RB, Payghan S, Deepeshwar S. Changes in Lung Function Measures Following Bhastrika Pranayama (Bellows Breath) and Running in Healthy Individuals. *Int J Yoga*. 2019;12(3):233-239. doi:10.4103/ijoy.IJOY_43_18

8.	Rangasamy V, Thampi Susheela A, Mueller A, F. H. Chang T, Sadhasivam S, Subramaniam B. The effect of a one-time 15-minute guided meditation (Isha Kriya) on stress and mood disturbances among operating room professionals: a prospective interventional pilot study. *F1000Research*. 2019;8:335. doi:10.12688/f1000research.18446.1

9.	Coronavirus Recovery: Breathing Exercises. Accessed May 3, 2021. https://www.hopkinsmedicine.org/health/conditions-and-diseases/coronavirus/coronavirus-recovery-breathing-exercises

10.	Maric V, Mishra J, Ramanathan DS. Using Mind-Body Medicine to Reduce the Long-Term Health Impacts of COVID-Specific Chronic Stress. *Front Psychiatry*. 2021;12. doi:10.3389/fpsyt.2021.585952

11.	Rain M, Subramaniam B, Avti P, Mahajan P, Anand A. Can Yogic Breathing Techniques Like Simha Kriya and Isha Kriya Regulate COVID-19-Related Stress? *Front Psychol*. 2021;12. doi:10.3389/fpsyg.2021.635816

12.	Narayanan S, Tennison J, Cohen L, Urso C, Subramaniam B, Bruera E. Yoga-Based Breathing Techniques for Health Care Workers During COVID-19 Pandemic: Interests, Feasibility, and Acceptance. *J Altern Complement Med*. Published online April 9, 2021. doi:10.1089/acm.2020.0536

#### B3. DESCRIPTION OF RESEARCH PROTOCOL

##### A. Study Design – Overview, Methods, Procedures

**Overview**

This study is a waitlisted randomized controlled trial. We aim to assess the level of compliance for those learning the intervention and to evaluate the impact of the practice on neuropsychological and somatic outcomes using validated scales. Enrollment into the study will be ongoing until we are able to get a sufficient sample size as described in the “Statistical Consideration” section. Upon enrollment and randomization, the intervention group will be followed weekly for 3 weeks as well as on week 6. The waitlisted control group will be followed for 3 weeks as controls and will then learn the intervention and be followed for additional 3 weeks (total of 6 weeks).

**Enrollment**

We will recruit study participants from the pool of patients with Long Covid Symptoms who are referred to the [Sadhguru Center for a Conscious Planet](https://www.bidmc.org/research/research-by-department/anesthesia-critical-care-and-pain-medicine/research-centers/sadhguru-center?fbclid=IwAR29oQcAKAzOrw_XbxyhAV50wzGqNSwJLHarKCgpG_479xu-ZZ5eQh1jHWc) by [BIDMC Critical Illness and COVID-19 Survivorship Program](https://www.bidmc.org/centers-and-departments/pulmonary-critical-care-and-sleep-medicine/services-and-programs/critical-illness-survivorship-program) and other similar programs, and are interested in attending the online Breathing and Wellness webinar. Participants can also self-refer or refer other participants experiencing Long Covid Symptoms with evidence of diagnosis. Participants will be asked whether they would be interested in being contacted for semi structured individual interviews and/or a focus group discussion (FGD). Those that would not like to be contacted can still participate in the study.

This Breathing and Wellness webinar and weekly Review webinars are being conducted by the Sadhguru Center for a Conscious Planet on a routine weekly basis. The Sadhguru Center is a multidisciplinary research center at BIDMC Department of Anesthesia and is dedicated to enhancing consciousness, cognition and compassion through research education and outreach. The interventions used for this study (Isha Kriya, Simha Kriya and Nadi Shuddhi) are developed by the international non-profit, [Isha Institute for Inner Sciences](https://isha.sadhguru.org/us-en/isha-usa/), and are made available to be used worldwide, free of charge.

The Sadhguru Center team will share the study information and flyer with the potential participants via email. Patients will be able to attend the training webinar regardless of their choice to enroll or not in the study. Please see the study invitation email and flyer as part of the attached documents.

Interested participants can use a public study link to access a study specific REDCap database wherein they will be asked a few screening questions to ensure study eligibility. Eligible participants will then be directed to an electronic consent form.

**Procedure:**

**Randomization:**

Participants will be randomized into an intervention group (Group 1) and a waitlisted control group (Group 2) upon signing the electronic consent.

**Intervention:**

The intervention selected for this study consists of two yogic breathing practices (Simha Kriya and Nadi Shuddhi) and a guided meditation (Isha Kriya) which will be delivered through an online webinar. Participants will be asked to practice these interventions daily and attend a weekly review webinar for 3 weeks.

**Breathing and Wellness Webinar:**

In this webinar, participants will learn the following 3 practices.

Simha Kriya : a 3-minute energizing breathing practice to expand lung capacity and improve immunity, to be practiced twice daily.

<https://www.youtube.com/watch?v=lP1Y1bk1YgU>

Nadi Shuddhi - a gentle 4-minute breathing practice for creating mental balance and relaxation, to be practiced for a minimum of 4 minutes daily.

<https://www.youtube.com/watch?v=q5m6tMjcF8k>

Isha Kriya - a 15-minute guided meditation that incorporates the breath and the awareness to create mental clarity and health, to be practiced at least once (ideally twice) daily.

<https://www.youtube.com/watch?v=EwQkfoKxRvo>

**Review webinar:**

Participants will be encouraged to attend a weekly review webinar where they go through the guided practices again and can ask any questions they may have about the practices.

Study Participants will complete baseline and weekly surveys that can take up to 20 minutes and a weekly compliance questionnaire that takes less than 5 minutes to complete.

**Assessments:**

- The baseline survey includes: demographic details, brief medical history, covid history, yoga, meditation and breathing practice details along with the PSS (Stress), POMS (Profile of Mood States), SF12 (Quality of Life), Multidimensional Dyspnea Profile (breathing discomfort), Somatic Symptom Scale (physical symptoms), and Free Text for the participants to include any additional symptom changes.
- The compliance questionnaire, weekly questionnaires as well as a satisfaction survey will be administered as described in the table below.
- Qualitative Portion: Participants who enroll in the study will have the option to participate in semi-structured individual interviews and/or a focus group discussion (FGD). Between 15-20 participants from the waitlist groups will be requested to participate in the semi-structured interviews, and the same range of participants number will be requested for the intervention group. The interviews will take place between weeks 1 and 3 of the study (before crossing into intervention) for the waitlist group and will take place between weeks 4 and 6 (after intervention period) for the intervention group. We will have three FGD that combines participants from the waitlist and intervention groups (standard 5-8 participants). The total number of participants required for the qualitative portion will be between (35-64). The duration of the semi-structured interviews and the FGD will be approximately 60-90 min. Participants who conducted the semi-structured individual interviews can also participate in the FGD.

The semi-structured interviews and the FGDs will take place via Teams video conferencing and will be conducted by members of the study team. All discussions will be audio-recorded and transcribed.

**Proposed study procedure and timeline:**

| **Time Point** | **Group 1: Intervention** | **Group 2: Waitlisted Control** |
| --- | --- | --- |
| Baseline | Consent → Randomization→ Baseline Survey | Consent → Randomized→ Baseline Survey |
| Intervention Training | Attending the Breathing and Wellness webinar | No Intervention  Semi-Structured Interviews |
| After 1 week | Webinar Check questionnaire  Compliance questionnaire  POMS (Mood)  SS8 (Somatic Symptoms)  MDP (Dyspnea Profile)  Free Text  Attending Review Webinar | POMS (Mood)  SS8 (Somatic Symptoms)  MDP (Dyspnea Profile)  Free Text  Semi-Structured Interviews |
| After 2 weeks | Compliance questionnaire  POMS (Mood)  SS8 (Somatic Symptoms)  MDP (Dyspnea Profile)  Free Text  Attending Review Webinar | POMS (Mood)  SS8 (Somatic Symptoms)  MDP (Dyspnea Profile)  Free Text  Semi-Structured Interviews |
| After 3 weeks | Compliance questionnaire  PSS (Stress)  POMS (Mood)  SF12 (Quality of Life)  SS8 (Somatic Symptoms)  MDP (Dyspnea Profile)  Semi-Structured Interviews  Free Text  Attending Review Webinar | POMS 37 (Mood)  PSS (Stress)  SF12 (Quality of Life)  SS8 (Somatic Symptoms)  MDP (Dyspnea Profile)  Free Text  Semi-Structured Interviews |
| Cross Over |  | Cross Over: Attending the Breathing and Wellness webinar |
| After 4 weeks | Semi-Structured Interviews |  |
| After 5 Weeks | Semi-structured Interviews | Compliance questionnaire  POMS (Mood)  SS8 (Somatic Symptoms)  MDP (Dyspnea Profile)  Free Text  Attending Review Webinar |
| After 6 Weeks | Compliance questionnaire  PSS (Stress)  POMS (Mood)  SF12 (Quality of Life)  SS8 (Somatic Symptoms)  MDP (Dyspnea Profile)  Free Text  Satisfaction Survey  Focus Group Discussion | Compliance questionnaire  PSS (Stress)  POMS (Mood)  SF12 (Quality of Life)  SS8 (Somatic Symptoms)  MDP (Dyspnea Profile)  Free Text  Satisfaction Survey  Attending Review Webinar  Focus Group Discussion |

Compliance is defined as completing 60% (4 days/week or more) of the activity that is prescribed in the protocol. This means that at least one of the three practices (Isha Kriya, Nadi Shuddhi, or Simha Kriya) must be completed once a day for a minimum of four days. Compliance data will be collected using a weekly activity log and will be used in data analysis.

For participants who have incomplete responses or are lost to follow up after consenting, the study team will reach out to them via either email or phone to remind them to complete the survey or withdraw if not interested. A phone call would also provide the opportunity to share reasons for not participating or withdrawing from the study. Those that continue to remain unresponsive will receive information about our Personalized Wellness Program after the completion of their study duration, along with those that have successfully completed the entire study.

Further, participants who agreed to participate in the semi-structured interview or FGD with the study team will also receive a phone call a day (+/- 7 days) after the initial scheduling email has been sent, additionally in concurrence with any follow-up emails to encourage them to schedule their appointment. They may also receive a phone call a day (+/- 3 days) before their scheduled interview appointment to remind them of their appointment and build rapport with the participant. If the participants misses their scheduled appointment, they may be contacted to reschedule.

The study team will review the “incomplete” surveys on REDCap for the consented participants and mark them as “complete” if the participants have answered all the required questions but failed to submit the survey. This is because the “Submit” button on REDCap is easy to miss and this feature cannot be modified in REDCap. The study team will also edit survey responses if email addresses are entered incorrectly into REDCap (e.g. gmmail.com instead of gmail.com). This will help prevent losing study participants who have answered the survey questions and are willing to continue their participation in the study.

In addition to the surveys, study participants will also receive instructional handouts and video links for guided practice. **Please See the attached instructional handout.**

Those who are not interested in the study or not eligible to participate, will receive a link to sign up for the Breathing and Wellness webinar. This is to make sure all Long Covid patients are offered the opportunity to participate in the webinars and learn the breathing and meditation practices even if they do not participate in the study.

**Questionnaire**

The following questionnaires are incorporated into the study and will be administered to the study participants at the aforementioned time points.

*Baseline demographics, medical history and yoga experience:*

This survey aims at collecting demographic details such as age, gender, race and ethnicity about the study participant. In addition, the research team is interested in collecting information about medical history (including Covid history) and previous yoga, meditation and breathing exercise experience and its duration and expertise level. The information collected will enable sensitivity analysis and adjust for any confounding effects either of these variables might have on the final study outcomes.

*Webinar Check Questionnaire:*

This questionnaire is added to make sure participants have completed the intervention webinar before they complete the follow up surveys. Questions in this questionnaire include queries on first webinar completion and the date of completion. If participant did not complete the first webinar, a link would be provided for them to review the webinar before proceeding ahead with the study.

(Revised questionnaire attached for further review)

*Compliance questionnaire:*

Defined as the study’s key primary outcome, the weekly compliance questionnaire is a tool which helps the participants to keep track of their activities each week. This enables the study team to measure compliance and protocol adherence by the participants by collecting information on their routine activity practiced, its frequency and duration.

*Perceived Stress Scale (PSS)*

PSS is defined as the key secondary outcome for this study and is a 10-question validated instrument that assesses stress. Participants are asked to rate on a scale of 0 (never) to 4 (very often) how often they agree with various statements. Items from each of the 10 questions are then summed to create a total perceived stress score.

*Profile of Mood States (POMS)*

This is a short validated survey used to measure six different dimensions of mood swings over a period of time. These include: Tension or Anxiety, Anger or Hostility, Vigor or Activity, Fatigue or Inertia, Depression or Dejection, Confusion or Bewilderment. A five-point scale ranging from "not at all" to "extremely" is administered and scores for each dimension contributes to calculation of positive and negative subscales in conjunction to total mood disturbance.

*Quality of Life Survey (SF12 )*

SF12 is a validated self-reported measure of the impact of health on everyday quality of life. It evaluates domains of physical activities, social activities, usual role activities, bodily pain, general mental health, vitality and general health perception.

*Multidimensional Dyspnea Profile (MDP)*

MDP is a validated scale to assess the overall breathing discomfort, sensory qualities and emotional responses using 8 questions. It is not intended for a particular activity and can be used during rest, activity or during clinical care. For assessing Long Covid patients, the first three parts (breathing discomfort and sensory qualities) are commonly used.

*Somatic Symptom Scale 8 items (SS8)*

SS8 is a validated brief questionnaire to assess common somatic symptoms including pain, shortness of breath, dizziness, fatigue and trouble sleeping. Participants are asked to rate on a scale of 0 (not at all) to 4 (very much) how often they agree with various statements. Items from each of the 8 questions are then summed to create a total perceived stress score.

*Free Text:*

This field is included for the participants to mention any additional symptoms or changes that are not covered by the standard questionnaires.

*Qualitative Interviews:*

The semi-structured individual interviews will allow us to gain rich, descriptive information about the participant's experience with the long COVID symptoms, treating physicians, and the effect of the provided practices on their symptoms. The focus group interviews will be semi-structured to ensure a systematic and flexible approach to data collection, allowing us to gain rich information about the participant's general experience with the current study and what matters to them as a Long COVID patients.

The interviews will cover the following main topics: (1) Perceptions of the participants on their long COVID symptoms, (2) Perceptions of the participants on their treating physicians (3) the effect of the provided meditation practices on their long COVID symptoms (4) and what matters to the Long COVID patients as well as their general experience as participants in the current study.

*Satisfaction Survey:*

Will be implemented at the end of the study to collect information about the experience of the subjects through the study and receive their feedback.

**Please see the survey details in the attached “Study Questionnaire” attachment.**

**Compensation for participating in the study:**

Participants will receive a $25 gift card upon completion of the study.

### B. Statistical Considerations

**Sample Size Justification:**

In this randomized pilot study, we are assessing compliance and the impact of the guided meditation practices on neuropsychological and somatic outcomes in study participants over a period of 3 weeks, using validated questionnaires. Our primary endpoint is observing a significant change in Perceived Stress Scale (PSS) between the control group and the meditation group.

We will be assuming that the variance is not equal in the study group before and after intervention. From previous studies, an average PSS score of 16 and standard deviation of 6. 25% off baseline score is considered a cut-off point for detecting significant change in PSS scores. Our effect size for this study will be 25%. A sample size of 48 - 74 subjects (24 – 37 subjects per study group) yields 80% power to detect a difference in neuropsychological outcomes in the cohort before and after the intervention. This sample size assumes the test used to compare the study cohort on the primary endpoint will be the independent t-test at a two-sided 0.05 level of significance.

To allow for 30-35% participant drop-out/loss to follow-up in this study, a total sample of 68 – 114 participants (34 – 54 subjects per study group) should be enrolled (42 - 55% increase) to see significant results.

Based on these considerations, we will recruit a minimum of 68 participants (34 per group) for this study.

| Mean PSS Control Group | Mean PSS Meditation Group | Power | Total Sample Size | Total Sample Size After 30% attrition rate |
| --- | --- | --- | --- | --- |
| 13 | 8 (Seen in previous meditation studies) | 80% | 48 | 68 |
|  |  | 90% | 64 | 90 |
| 13 | 9(25% effect size) | 80% | 74 | 105 |
|  |  | 90% | 98 | 139 |

***Data Analysis:***

Baseline characteristics of participants will be recorded. Continuous data will be presented as means ± standard deviation or median (interquartile range) depending on the distribution of the data and assess with a parametric or non-parametric t-test, as appropriate. Normality will be assessed with the Shapiro-Wilk test. Categorical data will be presented as frequencies and proportions and assessed with a chi-square or Fisher’s exact test. SAS 9.4 (SAS Institute Inc., Cary, NC) will be used for all analyses with two-sided p-values < 0.05 considered statistically significant.

***Analysis of Aim 1a:*** Trial feasibility and adherence will be assessed using data from how often participants in the meditation group complete their daily meditation sessions. This would be reported as the proportion of completed meditation sessions out of the total required per protocol.

***Analysis of Aim 1b*:** Given the continuous nature of the scales used to assess anxiety and depression, differences between groups will be assessed with a parametric or non-parametric t-test. Unadjusted and adjusted linear regression may be utilized in order to account for differences that persist. Our primary analyses will be assessed using intention-to-treat principles. In order to assess whether there are differences between baseline and at six weeks within a study group and between the study groups, we will employ the use of non-parametric paired and independent t-tests as appropriate.

An interim-analysis will be performed on the primary endpoint when 50% of patients have been randomized and have completed the 3 weeks follow-up. The interim-analysis is performed by an independent statistician, blinded for the treatment allocation. The statistician will report to the independent DSMC [data and safety monitoring committee]. The trial will not be stopped in case of futility, unless the IRB during the course of safety monitoring devices otherwise.

**Analysis of Aim 3:** Qualitatively, we will conduct between 30-40 semi-structured individual interviews. 15-20 participants from the waitlist group and 15-20 participants from the intervention group. The study will include one focus group with 5-8 participants. We will use a content and narrative analysis approach to analyze the collected data from the semi-structured individual interviews and the focus group discussion**.**There will be a review of the collected data starting with reading transcripts, open coding, and memos. Then data reduction will be made through codebook development and coding. Finally, we will construct categories, i.e., level 1 and level 2, in an iterative process and data interpretation. All the above will be done manually.

### C. Subject Selection

**Inclusion Criteria:**

- Age between 18 and 70 years
- Interested in attending the online Breathing and Wellness webinar.
- Laboratory-confirmed (PCR) history of Covid-19 infection.
- Long Covid condition diagnosis by a physician.
- Able to read and comprehend English.
- Currently residing in the United States.

**Exclusion Criteria:**

- Any medically limiting diagnosis that prevents a patient from doing the intervention or completing the assessments as determined by the PI such as severe Major Depression (under medication), Schizophrenia, Bipolar disorder.

Subjects will be enrolled without regards to race, gender, or vulnerable category status.

#### B4. POSSIBLE BENEFITS

We cannot guarantee any direct benefit to study participants. Insights gained from this trial may allow us to better understand the association between Yogic Breathing and Meditation and Long Covid symptoms and propose an approach to combat it. If successful, results of this trial could be used to inform other studies.

#### B5. POSSIBLE RISKS AND ANALYSIS OF RISK/BENEFIT RATIO

***Potential physical risks of the study interventions:*** The interventions offered in this study are simple and only involve Yogic Breathing and Guided Meditation practices. As such, there are no physical risks expected with these interventions. Similarly, we do not anticipate any risk of physical harm to any of the research subjects as a result of the assessment questionnaires used in this study.

***Potential psychological risks****: It is possible that there could be psychological or emotional risk to subjects in this study if they were to become aware of their performance in assessments, semi-structured individual interviews and the FGD, more so than they would be if they were not participating in the study. Subjects will not be given the scores of their assessments, but if they were to become aware of a decline in their performance this could be stressful or disconcerting to the subject. If, for any reason, participants indicate that they are a threat to themselves during qualitative interviews, the following steps will be followed:*

General plan:

A licensed clinician will be available by page or cellphone at all times and will respond immediately to any messages regarding a potential participant who may be incrisis.

Participants that are deemed as an immediate threat to themselves by bringing up suicidal ideation and/or thoughts of self-harm such as: life is not worth living, they are better off dead, or thoughts of harming or killing themselves will be immediately followed up with by a clinician who may either join the interview call or contact the participant separately while the participant is engaged with the study team. In this scenario, the clinician will advise the participant to go to the emergency room for urgent attention.

If it is determined that the participant is not agreeable to do as advised, then the following measures will be taken:

1. We will obtain information about where the participant is located.
2. 911/Authorities will be contacted and asked to check on the participant
3. The study team will stay on the call with the participant until help arrives.

*If the participant is* ***not*** *deemed as an immediate threat to themselves:*

A clinician will follow-up with the participant with crisis resources such as getting 24-hour emergency and crisis support.

In either situations, following measures will be taken:

1. The PI will also be notified immediately about the event.
2. The IRB would also be informed of the deviation within 5 working days of the PI’s awareness of the incident.

***Risk of breach of confidentiality:*** *As with any research study, there is a small risk associated with a breach of confidentiality. All research staff for this trial have been extensively trained in how to handle confidential data and how to minimize the possibility of this risk. To mitigate this risk, electronic data and that on paper will be kept in confidential, locked file cabinets and secure offices and/or on password-protected computers. Subjects will also be assigned a unique study ID to minimize this risk.*

**Risk/Benefit Ratio:** The intervention has no expected risks and the knowledge gained could potentially lead to studies that focus on improvement of psychological and somatic symptoms in the larger Long Covid patient population as well as in other conditions that accompany similar symptoms. Research team members will attempt to mitigate any of the possible risks described above; therefore, we believe the risk/benefit ratio is in keeping with allowing conduct of the trial.

#### B6. RECRUITMENT AND CONSENT PROCEDURES

**Recruitment**

We will recruit study participants from the pool of patients with Long Covid Symptoms who are referred to the [Sadhguru Center for a Conscious Planet](https://www.bidmc.org/research/research-by-department/anesthesia-critical-care-and-pain-medicine/research-centers/sadhguru-center?fbclid=IwAR29oQcAKAzOrw_XbxyhAV50wzGqNSwJLHarKCgpG_479xu-ZZ5eQh1jHWc) through [BIDMC Critical Illness and COVID-19 Survivorship Program](https://www.bidmc.org/centers-and-departments/pulmonary-critical-care-and-sleep-medicine/services-and-programs/critical-illness-survivorship-program) and other similar programs and are interested in attending the Breathing and Wellness webinar. Participants can also self-refer or refer other patients experiencing Long Covid symptoms with evidence of diagnosis.

The Sadhguru Center team will share the study information and flyer with the potential participants via email. This recruitment email consists of a public survey redcap link, where participants can confirm their interest in the study and get the detailed information of the study procedures. If they confirm interest in study after receiving the email, they will be directed by REDCap to a page where they will be asked a few screening questions to ensure study eligibility. Participants that are self-referred or referred by current participants can reach out to the Sadhguru Center team for the study’s REDCap link. Those who are eligible and willing to participate in the study will be directed to an electronic consent form.

Regarding the qualitative recruitment, only participants who prospectively agree for the semi-structured 1:1 interviews will receive further directions about the interview.

Additionally, interested participants can directly access the study’s REDCap link which would be made available on several platforms (e.g.: Sadhguru Center for a Conscious Planet’s official website, [BIDMC Critical Illness and COVID-19 Survivorship Program](https://www.bidmc.org/centers-and-departments/pulmonary-critical-care-and-sleep-medicine/services-and-programs/critical-illness-survivorship-program)’s official website, and other media outlets).

Patients will be able to attend the training webinar regardless of their choice to enroll or not in the study. Participants, who access the redcap link and show interest in attending the webinar but not participate in the study, can reach out to Sadhguru Center for a Conscious Planet and request for access to webinar’s only.

***Please see the invitation email and flyer attached***

**Consent**

Electronic Consent will be obtained through REDCap, following a few screening eligibility questions to ensure study eligibility in willing participants. The study procedures will be described in detail, including the perceived risks and benefits. The electronic consent will emphasize that participation is completely voluntary.

In addition, we will provide contact information for the study team to ensure that all of the participant’s questions are answered prior to providing consent. Electronic consent will then be obtained from willing participants prior to initiation of any study procedures for both the quantitative and qualitative data collection.

***Please see the attached consent form.***

**Subject Protection**

It is unlikely that participants will be vulnerable to coercion or undue influence in this study. Participants will be informed that their decision to participate or not to participate is entirely voluntary. Participants will have the ability to discontinue their participation at any time.

## B7. STUDY LOCATION

**Privacy**

All efforts will be taken to ensure participant privacy. All study interactions will be completed electronically. Throughout the study, only the minimum required information will be collected, assuring participant privacy during the study protocol. In the case of incomplete surveys and email entry errors, survey responses will need to be reviewed manually and edited to be marked as complete and to ensure accurate email entry for sustained communication with participants.

Data collection will occur only on password-protected computers secured by the BIDMC firewall or in REDCap directly. Data collected will be limited to only the minimum necessary to accomplish the stated research purposes.

**Physical Setting**

Electronic consent will be obtained from eligible participants. Study procedures can be completed by patients privately in their home. Follow up assessments will occur via online surveys.

Data will be stored on password-protected computers behind the BIDMC firewall or in REDCap directly, which is accessible to only the study team. Study investigators have been trained to maintain PHI in a secure and compliant manner

The FGDs will be conducted via Teams. It will be recorded and saved on a password-protected audio recorder. The recorded FGD will be deleted immediately after transcription.

## B8. DATA SECURITY

All electronic data will be stored in REDCap and/or on password-protected servers behind the BIDMC firewall. Any data collected on paper will be stored in locked file cabinets or in secure offices. All data would directly be entered into the REDCap database.

Each subject will be assigned a study-specific ID number. A crosswalk between the participants and their study IDs will be maintained on password-protected computers by members of the research team. At no time will this crosswalk be shared outside the study team.

All quantitative data would be directly entered into the REDCap database and at the completion of the study all identifiers will be deleted and data analysis will occur using only the participant's study ID, to further ensure confidentiality of their responses.

Qualitative data: All interviews and FGDs will be recorded and saved on password-protected computers secured by the BIDMC firewall by members of the research team. All recordings will be deleted after data analysis. They will not be shared outside the study team. The collected information (transcripts) will be saved in a locked disk at the PI’s personal office. The study progress reports, and the ongoing analysis results, will be shared between the PI and the research team.

## B9 Multi-Site Studies

**This is a Single Center Study**

Is the BIDMC the coordinating site? ☐ Yes	☐ No

Is the BIDMC PI the lead investigator of the multi-site study? ☐ Yes	☐ No

## B10 Dissemination of Research Results

*Please explain whether you will be able to thank subjects and provide research results and, if so, how this will be accomplished. If you do not think this is feasible, appropriate or applicable to this research, please specify why.*

Participants will be thanked for their time spent throughout the study. We do not intend to keep participant contact information after the study has been completed. Therefore, we will not be able to directly contact them regarding study results.

Results will be used for presentations and grants. It is possible that they will be available via publication. Final results will also be shared with Isha Foundation.
