# Supplementary material for: Web-Based Isha Kriya Meditation and Breathwork in Long COVID: Mixed Methods Randomized Controlled Pilot Study
Source: J Med Internet Res. 2026 Nov 12;28:e89631. doi: 10.2196/89631 (PMC13758968; doi:10.2196/89631)
Supplement: Supplementary file 6 [file jmir-v28-e89631-s002.docx]

*The below YouTube links are the demonstration videos for the three meditation and breathing practices taught in the Breathing and Wellness program.*

1. Isha Kriya: <https://youtu.be/EwQkfoKxRvo?si=ocbK-dzLmaaQ0STQ>
2. Nadi Shuddhi : <https://youtu.be/q5m6tMjcF8k?si=j_aPPTK6owZ_ZsvW>
3. Simha Kriya: <https://youtu.be/lP1Y1bk1YgU?si=hGFiyrY9n1FWl2Zg>
