# Supplementary material for: Web-Based Isha Kriya Meditation and Breathwork in Long COVID: Mixed Methods Randomized Controlled Pilot Study
Source: J Med Internet Res. 2026 Nov 12;28:e89631. doi: 10.2196/89631 (PMC13758968; doi:10.2196/89631)
Supplement: Supplementary file 7 [file jmir-v28-e89631-s003.docx]

**Preparation and setting.** Participants were instructed to avoid eating a meal just before the session and to prepare for webinars by sitting cross-legged or on a chair in a quiet, private space within full view of the instructor.

**Instructor training program.** Training proceeded in three phases: (1) a preparatory phase in which trainees reviewed program materials and the long-COVID-specific curriculum; (2) a required 5-week training phase in which trainees observed multiple live sessions led by experienced instructors and completed a mock teaching session, evaluated by another trained instructor using a standardized rubric; and (3) a supported teaching phase in which new instructors led sessions under oversight before teaching independently. Instructors were required to be rated as ready to teach independently, based on this standardized evaluation, before doing so.

**Long-COVID-specific curriculum content.** The curriculum explicitly addressed the substantial variability of long COVID, including its fluctuating symptom course both between individuals and within the same individual over time, and emphasized individualized accommodation rather than a fixed symptom-response protocol. Training included a structured reference guide covering accommodations for commonly reported symptoms (including dyspnea, fatigue, dizziness, cognitive difficulties, and post-exertional symptom worsening) and practice-specific troubleshooting guidance for difficulties reported by this population (e.g., breathlessness or throat discomfort during Simha Kriya, nasal congestion during Nadi Shuddhi). Instructors documented participant-reported symptoms affecting practice completion in a shared tracking system and were instructed to escalate any symptom or question outside their training to the study's medical co-investigators via a structured communication protocol.
