# Supplementary material for: Web-Based Isha Kriya Meditation and Breathwork in Long COVID: Mixed Methods Randomized Controlled Pilot Study
Source: J Med Internet Res. 2026 Nov 12;28:e89631. doi: 10.2196/89631 (PMC13758968; doi:10.2196/89631)
Supplement: Supplementary file 8 [file jmir-v28-e89631-s004.docx]

**Compliance Questionnaire**

1) In the past week, how many days did you practice Isha Kriya? (Drop Down Menu 1 to 7)
2) In the past week, how many days did you practice Nadi Shuddhi? (Drop Down Menu 1 to 7)
3) In the past week, how many days did you practice Simha Kriya?(Drop Down Menu 1 to 7)
4) In the past week, how many days did you practice Simha Kriya **Twice**? (Drop Down Menu 1 to 7)
5) During the past week, did you attend the Breathing and Wellness **Review session**?
   a) Yes
   b) No
6) If No, to Review session,
   c. Why did you not attend the Review session?
      i. I did not know about it
      ii. I did not have time
      iii. I did not think it would be useful
      iv. I prefer not to specify
