# Supplementary material for: Web-Based Isha Kriya Meditation and Breathwork in Long COVID: Mixed Methods Randomized Controlled Pilot Study
Source: J Med Internet Res. 2026 Nov 12;28:e89631. doi: 10.2196/89631 (PMC13758968; doi:10.2196/89631)
Supplement: Supplementary file 9 [file jmir-v28-e89631-s006.docx]

**Semi-structured individual interviews questions:**

Group 1 (Intervention):

**Perceptions of Participants Symptoms**

1. Will you walk us through your experience with COVID disease?
   a. Prob:
      i. How did you find out that you are COVID positive?
      ii. How did you feel about being COVID positive?
      iii. b- How did you find out that you are a long COVID patient?
      iv. What was it like when you did not know what was going on?
      v. Whom did you talk to?

vi. What was it like being diagnosed as a long COVID patient?

2. Can you tell us about your life before being diagnosed as a Long COVID patient? How is it different from now?
   a. Prob: daily living activities /mood changes/ communication and relationships (with family and friends) with other /work
3. As a long COVID patient, what are your biggest fears and why?
4. Will you share with us what are you doing to cope with your long COVID symptoms?
5. What would you advise others (newly diagnosed patients, family members, doctors)?

Prob: b- what was helpful and what wasn’t?

**For patients with Brain Fog only:**

1-What is it like for you to not be able to remember your COVID journey? How does that make you feel?

**Perceptions of Physicians**

1. Would you mind sharing with us your overall experience with your long COVID treating physician?
   a. Prob:
i. Did you have adequate time with them?
ii. Was your doctor listening to your complains?
iii. Were they able to address all your queries/complains?
2. What is your experience with your initial physician visit?
3. From your point of view, what should the treating physician do to improve the quality of the provided medical service for patients with Long COVID symptoms?
4. What other changes do you suggest for the health care system to better support Long COVID patients?

**Intervention Interview Questions:**

1. Would you tell us why you chose to enroll in the mindfulness practices as a way of managing your long COVID symptoms? what did you hope to find out of this? What did you expect?
2. How did you feel after the very first class or workshop?
3. How did you feel after the very first time you learned the practices? How did you feel during the practice?

4. Among the three practices, which one did you prefer and why?

a. Prob: How often do you do the practices?

b. What were your challenges?

5. How did the practices help you to cope with your long COVID symptoms?

Group 2 (Waitlisted Control):

**Perceptions of Participant Symptoms**

**1-**Will you walk us through your experience with COVID disease?

Prob: i- How did you find out that you are COVID positive?

ii-How did you feel about being COVID positive?

iii- How did you find out that you are a long COVID patient?

iv.What was it like when you did not know what was going on?

v.Whom did you talk to?

vi. What was it like being diagnosed as a long COVID patient?

1. Can you tell us about your life before being diagnosed as a Long COVID patient? How is it different from now?
   a. Prob: daily living activities /mood changes/ communication and relationships (with family and friends) with other /work
2. As a long COVID patient, what are your biggest fears and why?
3. Will share with us what are you doing to cope with your long COVID symptoms?
4. What would you advise or share with a newly diagnosed long COVID patients, family, and physicians?

For patients with Brain Fog only:

1-What is it like for you to not be able to remember your COVID journey? How does that make you feel?

**Perceptions of Physicians**

6. Would you mind sharing with us your overall experience with your long COVID treating physician?
   a. Prob:
iv. Did you have adequate time with them?
v. Was your doctor listening to your complains?
vi. Was they able to address all your queries/complains?
7. What is your experience with your initial physician visit?
8. From your point of view, what should the treating physician do to improve the quality of the provided medical service for patients with Long COVID symptoms?
9. What other changes do you suggest for the health care system to better support Long COVID patients?

**Intervention Interview Question:**

10. Would you tell us why you chose to enroll in the mindfulness practices as a way of managing your long COVID symptoms?

**Focus Group Discussion Interview Questions:**

Focus Group Discussion Questions:

1- Will you share with us how your experience was with the waitlist /intervention group?

Prob: - what do you think about it

-What should be changed

- what should be added

2- What is your take away from this experience?

Prob: will you continue doing the practices?

3- As a long COVID symptoms patient, what matters the most to you?
4- What would you advise to improve our program and why?
5- Will you recommend a friend or someone you know having long COVID symptoms joining our program? Yes/No and why?
