# Supplementary material for: Web-Based Isha Kriya Meditation and Breathwork in Long COVID: Mixed Methods Randomized Controlled Pilot Study
Source: J Med Internet Res. 2026 Nov 12;28:e89631. doi: 10.2196/89631 (PMC13758968; doi:10.2196/89631)
Supplement: Supplementary file 10 [file jmir-v28-e89631-s007.docx]

**Table S1. Raw Outcome Scores at Baseline and Week 3 in Intervention and Waitlist Control Groups (n=185).**

|  | **Intervention (n=94)** | | **Waitlist (n=91)** | |
| --- | --- | --- | --- | --- |
|  | **Baseline** | **Week 3** | **Baseline** | **Week 3** |
|  | mean (SD) | mean (SD) | mean (SD) | mean (SD) |
| PSS | 20.3 (7.2) | 19.7 (6.7) | 20.3 (6.5) | 20.9 (7.0) |
| POMS |  |  |  |  |
| Total | 34.0 (22.3) | 21.0 (23.4) | 36.4 (23.3) | 35.3 (27.0) |
| Positive | 15.3 (5.3) | 18.1 (6.5) | 14.9 (4.8) | 14.2 (6.4) |
| Vigor | 4.1 (3.3) | 5.6 (4.3) | 3.7 (2.4) | 3.4 (3.6) |
| Esteem-related Affect | 11.2 (2.9) | 12.6 (3.2) | 11.2 (3.0) | 10.8 (3.7) |
| Negative | 49.3 (18.7) | 39.1 (19.5) | 51.3 (20.2) | 49.5 (22.1) |
| Tension | 10.1 (5.2) | 8.2 (6.0) | 10.4 (5.4) | 10.3 (5.5) |
| Depression | 10.3 (5.0) | 8.3 (5.0) | 10.7 (5.7) | 10.2 (6.1) |
| Anger | 6.7 (4.4) | 4.9 (3.9) | 7.0 (4.7) | 6.5 (4.8) |
| Fatigue | 14.0 (4.2) | 10.9 (4.9) | 14.3 (4.0) | 14.0 (4.7) |
| Confusion | 8.3 (4.5) | 6.9 (4.1) | 8.9 (4.3) | 8.4 (5.2) |
| SF12 |  |  |  |  |
| PCS12 | 32.7 (8.3) | 34.6 (8.3) | 31.1 (7.5) | 32.4 (7.3) |
| MCS12 | 40.0 (10.4) | 40.4 (9.9) | 38.1 (9.1) | 38.1 (9.0) |
| MDP |  |  |  |  |
| Immediate Perception domain | 25.6 (13.0) | 22.8 (13.5) | 26.9 (12.7) | 30.6 (12.6) |
| A1 Breathing discomfort | 4.7 (2.3) | 4.5 (2.1) | 5.2 (2.2) | 5.3 (1.9) |
| Sensory dimension | 20.9 (11.3) | 18.3 (11.7) | 21.6 (11.2) | 25.3 (11.0) |
| SS8 | 15.2 (5.1) | 12.6 (6.3) | 15.9 (5.6) | 15.1 (5.7) |

*PSS=Perceived Stress Scale. POMS=Profile of Mood States scale. SF-12 = Quality of Life questionnaire. PCS12=physical health composite. MCS12=mental health composite. MDP=Multidimensional Dyspnea Profile. SS8=Somatic Symptom Burden – 8 item scale. The analytic sample includes 185 participants who provided at least one PSS measurement. Three randomized participants had no PSS data at any time point and were therefore not analyzable in the mixed-effects models.*

**Table S2. Alternative thresholds per week for adherence classification among participants with long COVID by arm**

| **Arm** | **≥3 days** | **≥ 4 days** | **≥ 5 days** |
| --- | --- | --- | --- |
| Intervention (n=30) | 83.8% | 60% | 50% |
| Waitlist (n=28) | 60.7% | 50% | 32.1% |
| All participants (n=58) | 72.4% | 55.2% | 41.4% |

*Notes: Percentages indicate proportion of participants in each group that met thresholds of ≥ 3 days, ≥ 4 days, and ≥ 5 days of practice per week during the active intervention period. Participants were included if they had complete data for all three weeks of the active intervention week (n=58). The original practice threshold was defined as completing at least one of the three intervention practices (Isha Kriya, Nadi Shuddhi, Simha Kriya) on* *≥ 4 days per week, in all three of a participant's active-intervention weeks.*

**Table S3. Baseline demographic and clinical characteristics of retained versus non-retained participants (Week 6 Completion Status; n=185)**

| **Characteristic** | **Not Retained** N = 117*^1^* | **Retained** N = 68*^1^* | **p-value***^2^* |
| --- | --- | --- | --- |
| Randomization |  |  | 0.5 |
| Intervention | 62 (53%) | 32 (47%) |  |
| Waitlist | 55 (47%) | 36 (53%) |  |
| **Age** |  |  | 0.3 |
| 18-29 | 14 (12%) | 4 (5.9%) |  |
| 30 -39 | 30 (26%) | 13 (19%) |  |
| 40-49 | 35 (30%) | 20 (29%) |  |
| 50-59 | 24 (21%) | 16 (24%) |  |
| 60-69 | 13 (11%) | 14 (21%) |  |
| 70+ | 1 (0.9%) | 1 (1.5%) |  |
| **Gender** |  |  | 0.4 |
| Female | 89 (76%) | 55 (82%) |  |
| Male | 25 (21%) | 12 (18%) |  |
| Other | 3 (2.6%) | 0 (0%) |  |
| **Race** |  |  | **0.038** |
| White | 93 (79%) | 65 (96%) |  |
| Asian | 7 (6.0%) | 2 (2.9%) |  |
| Black or African American | 8 (6.8%) | 0 (0%) |  |
| Multi-Racial | 2 (1.7%) | 1 (1.5%) |  |
| Other | 3 (2.6%) | 0 (0%) |  |
| NA | 4 (3.4%) | 0 (0%) |  |
| **Ethnicity** |  |  | 0.3 |
| Hispanic or Latino | 4 (3.4%) | 3 (4.6%) |  |
| Not Hispanic or Latino | 108 (93%) | 62 (95%) |  |
| NA | 4 (3.4%) | 0 (0%) |  |
| **Outcomes** |  |  |  |
| PSS | 21 (7) | 20 (7) | 0.3 |
| TMD | 38 (23) | 30 (22) | **0.026** |
| SS8 | 16.1 (5.4) | 14.6 (5.1) | 0.065 |
| PCS12 | 32 (8) | 32 (7) | 0.9 |
| MCS12 | 38 (10) | 41 (9) | 0.087 |
| MDP_A1 | 4.92 (2.29) | 4.98 (2.24) | 0.9 |
| *^1^* n (%); Mean (SD) | | | |
| *^2^* Fisher’s exact test; Welch Two Sample t-test | | | |

*Note: Retained participants are those who completed up to the Week 6 study survey. Three participants were not included due to missing baseline data. PSS=Perceived Stress Scale. POMS=Profile of Mood States scale. SF-12 = Quality of Life questionnaire. PCS12=physical health composite. MCS12=mental health composite. MDP=Multidimensional Dyspnea Profile. SSS-8=Somatic Symptom Burden – 8 item scale.*

**Table S4. Baseline demographic and clinical characteristics of adherent versus non-adherent participants with long COVID, among those with complete 3-week adherence data (n=58)**

| **Characteristic** | **Non-adherent** N = 26*^1^* | **Adherent** N = 32*^1^* | **p-value***^2^* |
| --- | --- | --- | --- |
| **Randomization** |  |  | 0.6 |
| Intervention | 12 (46%) | 18 (56%) |  |
| Waitlist | 14 (54%) | 14 (44%) |  |
| **Age** |  |  | 0.8 |
| 18-29 | 2 (7.7%) | 1 (3.1%) |  |
| 30-39 | 5 (19%) | 6 (19%) |  |
| 40-49 | 8 (31%) | 10 (31%) |  |
| 50-59 | 5 (19%) | 10 (31%) |  |
| 60-69 | 6 (23%) | 5 (16%) |  |
| **Gender** |  |  | 0.5 |
| Female | 19 (76%) | 27 (84%) |  |
| Male | 6 (24%) | 5 (16%) |  |
| **Race** |  |  | >0.9 |
| Asian | 1 (3.8%) | 1 (3.1%) |  |
| Black or African American | 0 (0%) | 1 (3.1%) |  |
| White | 25 (96%) | 30 (94%) |  |
| **Ethnicity** |  |  | >0.9 |
| Hispanic or Latino | 1 (4.0%) | 2 (6.5%) |  |
| Not Hispanic or Latino | 24 (96%) | 29 (94%) |  |
| **Outcomes** |  |  |  |
| PSS | 19 (8) | 20 (6) | 0.7 |
| TMD | 26 (24) | 35 (20) | 0.13 |
| SS8 | 14.6 (5.0) | 14.6 (5.4) | >0.9 |
| PCS12 | 33 (8) | 32 (6) | 0.6 |
| MCS12 | 41 (8) | 40 (11) | 0.7 |
| MDP_A1 | 5.57 (2.35) | 4.55 (2.20) | 0.11 |
| *^1^* n (%); Mean (SD) | | | |
| *^2^* Fisher’s exact test; Welch Two Sample t-test | | | |

*Notes: Adherence was defined as those who reported completing at least one intervention practice (Isha Kriya, Nadi Shuddhi, or Simha Kriya) on ≥4 days/week in all three of a participant's active-intervention weeks.* *Those who were missing adherence data in any of these three weeks due to non-response or study withdrawal were excluded from this table.* *PSS=Perceived Stress Scale. POMS=Profile of Mood States scale. SF-12 = Quality of Life questionnaire. PCS12=physical health composite. MCS12=mental health composite. MDP=Multidimensional Dyspnea Profile. SSS-8=Somatic Symptom Burden – 8 item scale.*

**Table S5. Pooled analysis of secondary outcome changes during intervention and waitlist control's own active-intervention period (n=155)**

|  | **Adjusted Mean Difference^a^**  **(95% CI)** | **P-value^b^** | ***η^c^*** |
| --- | --- | --- | --- |
| PSS | -0.92 (-1.9 to 0.09) | .07 | 0.04 |
| POMS |  |  |  |
| TMD | -9.4 (-14 to -4.8) | **< .001** | 0.19 |
| Positive | 1.4 (0.16 to 2.7) | **.03** | 0.06 |
| Vigor | 0.88 (0.001 to 1.8) | **.05** | 0.03 |
| Esteem-related Affect | 0.60 (0.01 to 1.2) | **.05** | 0.05 |
| Negative | -8.0 (-12 to -4.2) | **< .001** | 0.20 |
| Tension | -1.8 (-2.9 to -0.64) | **.003** | 0.11 |
| Depression | -1.2 (-2.2 to -0.26) | **.01** | 0.08 |
| Angers | -1.4 (-2.2 to -0.57) | **.001** | 0.14 |
| Fatigue | -2.5 (-3.6 to -1.5) | **< .001** | 0.21 |
| Confusion | -1.2 (-1.9 to -0.46) | **.002** | 0.14 |
| SF12 |  |  |  |
| PCS12 | 3.1 (1.6 to 4.6) | **< .001** | 0.18 |
| MCS12 | 0.45 (-1.6 to 2.5) | .67 | 0.003 |
| MDP |  |  |  |
| Immediate Perception domain | -4.3 (-6.5 to -2.2) | **< .001** | 0.19 |
| A1 Breathing discomfort | -0.55 (-0.99 to -0.11) | **.01** | 0.08 |
| Sensory dimension | -3.8 (-5.7 to -1.9) | **< .001** | 0.18 |
| SS8 | -2.1 (-2.9 to -1.3) | **< .001** | 0.28 |
| *PSS=Perceived Stress Scale. POMS=Profile of Mood States scale. SF-12 = Quality of Life questionnaire. PCS12=physical health composite. MCS12=mental health composite. MDP=Multidimensional Dyspnea Profile. SS8=Somatic Symptom Burden – 8 item scale. Baseline scores were unavailable for 30 participants due to incomplete study surveys.*  *^a^Adjusted mean difference in change between baseline and endpoint. Baseline consists of baseline data from the intervention group and week 3 data from the waitlist group. Endpoints consist of week 3 data from the intervention group and week 6 data from the waitlist group.*  *^b^p-value for time effects*  *^c^ η² >= .14 indicates a large effect size, η² >= .06 medium effect size and η² >= .01 small effect size* | | | |

**Table S6.** **Association between adherence status and change in secondary outcomes during each arm's own active-intervention period, under complete-case, worst-case, and best-case missing-data assumptions**. **(n = 58)**

| **Outcome** | **Complete-case** **Estimate (SE), *P*** | **Worst-case** **Estimate (SE), *P*** | **Best-case** **Estimate (SE), *P*** |
| --- | --- | --- | --- |
| PSS | 1.04 (1.10), .347 | 0.72 (1.04), .492 | 0.15 (1.04), .889 |
| POMS TMD | −4.04 (5.04), .427 | −2.45 (4.82), .613 | −5.23 (4.76), .276 |
| SF-12 |  |  |  |
| PCS-12 | −1.13 (1.84), .542 | −0.50 (1.57), .754 | −1.01 (1.57), .521 |
| MCS-12 | 0.14 (2.51), .956 | −0.54 (2.18), .805 | 0.99 (2.17), .648 |
| MDP |  |  |  |
| Immediate Perception Domain | 1.12 (2.68), .679 | 0.56 (2.24), .805 | −1.01 (2.25), .656 |
| A1 (breathing discomfort) | 0.46 (0.53), .389 | 0.42 (0.45), .361 | −0.14 (0.46), .753 |
| Sensory Dimension | 0.61 (2.38), .800 | 0.11 (1.97), .955 | −0.97 (1.98), .628 |
| SSS-8 | −0.66 (0.87), .452 | −0.19 (0.81), .813 | −1.13 (0.80), .162 |

*Note. Change reflects each arm's own active intervention period (intervention: Baseline to Week 3; waitlist: Week 3 to Week 6), not a shared calendar timepoint. Estimates represent the adjusted mean difference in outcome change (adherent minus non-adherent), from linear mixed-effects models adjusted for age, gender, race, and ethnicity. Complete-case analysis is restricted to participants with complete 3-week adherence data (n=56–58 participants, depending on outcome); worst-case and best-case analyses apply the corresponding missing-data assumption to the full adherence-outcome population (n=103–106 participants, depending on outcome). No estimate reached statistical significance (all P>.05) under any scenario. Because adherence status is determined after randomization, these associations are exploratory and non-causal.*

**Table S7. Association between continuous adherence (mean weekly practice frequency) and change in secondary outcomes during each arm's own active-intervention period**

| **Outcome** | **Estimate (SE)** | ***P*** | **Partial η²** | ***n*** |
| --- | --- | --- | --- | --- |
| PSS | −0.28 (0.30) | .354 | .013 | 83 |
| POMS |  |  |  |  |
| TMD | −1.77 (1.27) | .169 | .030 | 83 |
| Positive Mood | 0.33 (0.38) | .393 | .011 | 83 |
| Negative Mood | −1.45 (1.02) | .160 | .032 | 83 |
| SF-12 |  |  |  |  |
| PCS-12 | 0.07 (0.45) | .874 | <.001 | 82 |
| MCS-12 | 0.02 (0.64) | .973 | <.001 | 82 |
| MDP |  |  |  |  |
| Immediate Perception domain | −0.57 (0.67) | .396 | .011 | 80 |
| A1 (breathing discomfort) | −0.14 (0.14) | .292 | .017 | 80 |
| Sensory Dimension | −0.44 (0.59) | .456 | .009 | 80 |
| SSS-8 | −0.56 (0.22) | **.015** | .086 | 83 |
| *Note. Estimates represent the adjusted change in outcome per additional day/week of practice (mean weekly frequency × time interaction), from linear mixed-effects models adjusted for arm, age, gender, race, and ethnicity. Negative estimates indicate favorable change (improvement) for all outcomes except POMS Positive Mood, SF-12 Physical Component, and SF-12 Mental Component, where positive estimates are favorable. n reflects participants with ≥1 responded adherence week and valid outcome data for that measure (larger than the complete-case population in Table S6, since this analysis does not require complete 3-week adherence data). Bold indicates P<.05. Because adherence is determined post-randomization, this association is exploratory and non-causal.* | | | | |
