# Supplementary material for: Web-Based Isha Kriya Meditation and Breathwork in Long COVID: Mixed Methods Randomized Controlled Pilot Study
Source: J Med Internet Res. 2026 Nov 12;28:e89631. doi: 10.2196/89631 (PMC13758968; doi:10.2196/89631)
Supplement: Supplementary file 11 [file jmir-v28-e89631-s008.docx]

**Adherence definition.** Adherence classification held in direction across alternative thresholds (72.4% at ≥3 days/week, 41.4% at ≥5 days/week, vs. 55.2% at the primary ≥4-day threshold), with the intervention arm remaining more adherent than the waitlist arm at every threshold (Table S2). Retained and non-retained participants differed at baseline only in race and total mood disturbance (retained participants were more often White, with lower baseline TMD), a caveat worth considering for generalizability (Table S3); adherent and non-adherent participants did not differ at baseline (Table S4), suggesting adherence was not driven by baseline severity.

**Efficacy.** A pooled model combining both cohorts' active-intervention periods (n=155) showed a similar overall effect with larger effect sizes than the primary model (Table S5), and a per-protocol analysis of Week 6 completers showed no significant differences from the primary mITT results (Table S6). Because adherence is determined post-randomization, all adherence-outcome associations below are secondary, exploratory, and non-causal. A complete-case comparison of adherent versus non-adherent participants showed no significant interaction with any outcome under complete-case, worst-case, or best-case assumptions (Table S7); direction was stable for mood, somatic symptom burden, physical health, and stress, but reversed under best-case assumptions for mental health and both MDP dyspnea measures. Since none of these reached significance under any assumption, we do not interpret this as evidence of a true effect, though it further underscores uncertainty in the dyspnea-adherence relationship given the extent of missing data. Modeling adherence as a continuous gradient showed directionally favorable associations across all nine outcomes, reaching significance only for somatic symptom burden (β=−0.56, *P*=.015, η²=.086; Table S8). This was not seen in the binary comparison above, possibly reflecting a true dose-dependent relationship. Given the consistent direction without consistent significance across analyses and the multiple comparisons, these exploratory findings are best treated as hypothesis-generating.
